# Modeling Softening Kinetics at Cellular Scale and Phytochemicals Extractability in Cauliflower under Different Cooking Treatments

**DOI:** 10.3390/foods10091969

**Published:** 2021-08-24

**Authors:** Ancuta Nartea, Pasquale Massimiliano Falcone, Luisa Torri, Babak Ghanbarzadeh, Natale Giuseppe Frega, Deborah Pacetti

**Affiliations:** 1 Department of Agricultural, Food and Environmental Sciences (D3A)—Marche University Polytechnical, Monte Dago, Via Brecce Bianche, 10-60131 Ancona, Italy; a.nartea@pm.univpm.it (A.N.); n.g.frega@univpm.it (N.G.F.); d.pacetti@univpm.it (D.P.); 2 University of Gastronomic Sciences, Piazza Vittorio Emanuele II, 9-12042 Pollenzo, Cuneo, Italy; l.torri@unisg.it; 3 Department of Food Science and Technology, University of Tabriz, Tabriz 51666-16471, Iran; babak.ghanbarzadeh@neu.edu.tr

**Keywords:** colored cauliflower, rheological properties, cell wall loosening, cell separation, tocopherols, sterols, boiling, steaming, sous-vide

## Abstract

The effects induced by heat on *Depurple* and *Cheddar* (*Brassica oleracea* L. var. botrytis) during boiling, steaming, and *sous-vide* were investigated to elucidate the role of the basic cellular elements in softening and extractability of sterols and tocopherols. With this aim, an elastoplastic mechanical model was conceptualized at a cell scale-size and validated under creep experiments. The total amount of the phytochemicals was used to validate multivariate regression models in forecasting. Boiling was the most effective method to enhance the softening mechanisms causing tissue decompartmentalization through cell wall loosening with respect to those causing cell separation, having no impact on the phytochemical extractability. *Sous-vide* showed the lowest impact on cell wall integrity, but the highest in terms of cell separation. Steaming showed an intermediate behavior. Tissue of the *Depurple* cauliflower was the most resistant to the heat, irrespectively to the heating technology. Local heterogeneity in the cell wall and cell membrane, expected as a plant variety-dependent functional property, was proposed as a possible explanation because sterol extractability under lower heat-transfer efficiency, i.e., steaming and *sous-vide*, decreased in *Depurple* and increased in *Cheddar* as well as because the extractability of sterols and tocopherols was greater in *Cheddar*.

## 1. Introduction

There is a significant amount of literature showing that thermal processing conducted to prepare vegetable foods for consumption may cause changes in polymer composition and interaction at different ranges of scale size (cell, tissue, and organ level) simultaneously, resulting in texture softening and, with different extent, in cell wall disassembling and phytochemical decompartmentalization. As reviewed by Ranganathan et al. [1] and Ling et al. [2], during heating, several microstructural factors having an influence on texture softening are widely recognized: the composition and interaction of the cellulose, hemicellulose, pectin assemblies at a cell wall and middle lamella level, thickness of cells, turgor pressure inside the cell, and finally, the cellular arrangement in plant tissue, which includes the organization of cells as groups and their size and shape. All these factors may change under heating, creating a sequence of responses over a wide range of scale sizes, which include loss of compartmentalization of the intracellular phytochemicals on a microscopic scale and texture softening on a macroscopic scale. The membrane disruption is considered the main factor causing firmness loss during thermal processing; cellulose and hemicelluloses behave as inert materials (the only change in cellulose during thermal processing is an increase in the degree of hydration), and pectin is a more chemically reactive polymer undergoing a variety of enzymatic and chemical modifications under heating.

As reviewed by Holland [3], the extent of decompartmentalization of vegetable tissue microstructure during heating affects the successive bioaccessibility of intracellular bioactive macromolecules during consumption. As a result, the vegetable nutritional value, as well as the health effects related to its consumption, depends not only on the amount of bioactive compounds present in the fresh matrix but also on the assembling and disassembling mechanisms that occur during thermal processing and that are strictly related to the time-temperature conditions experienced during heating [4,5,6,7,8,9,10].

Focusing the attention on cauliflower (*Brassica oleracea* L. var. botrytis), it displays a wide spectrum of bioactive compounds (e.g., carotenoids, tocopherols, glucosinolates, and phenols) [11,12,13,14,15] and drivers of health benefits that may work in synergism as suggested by Koss-Mikołajczyk et al. [16]. Previous research [5,17] emphasized that boiling and *sous-vide* cooking strongly reduced the levels of phenolic compounds (i.e., quercetin and kaempferol) and glucosinolates in white cauliflower (*Brassica oleracea* L. var. Alphina F1), whereas steaming determined an increase of their glucosinolates total contents [18]. Given that orange cauliflower (*Cheddar*) may be a good source of carotenoids and tocopherols, it has been proven that boiling, steaming, and *sous-vide* increased the carotenoids and tocopherols extractability in colored cauliflower [12,19]. Despite the cauliflower resulting in a rich source of phytosterol (310–400 mg/kg FW) [20] and having hypoglycemic and hypolipidemic activities (EFSA health claim), no data was found regarding the impact of heating on the level of these compounds.

The main tissues of the edible part of cauliflower inflorescences consist of parenchyma cells, the most of which remain meristematic at maturity, with a high cellular proliferation [21]. This density of living cells makes the cauliflower inflorescence an organ with a considerable proportion of compartmentalizing membranes, and therefore rich in proteins associated with bilayer of lipids, including sterols and tocopherols. Sterols and tocopherols are compartmentalized at the lowest level of the tissue hierarchy. Tocopherols are synthesized in the inner membrane of cytoplasmatic chloroplasts and then distributed between chloroplast membranes, thylakoids, and plastoglobules, where they exert their protective ability by scavenging and quenching reactive oxygen species and fat-soluble by-products of oxidative stress [22]. Free sterols and to an extent, steryl glycosides and acylated steryl glycosides, are incorporated into cell membranes, while the esterified sterols are present in the cytosol as storage forms of sterols [23,24]. As reviewed by Schirck et al. [24], molecular interactions in sterol-rich microdomains or another form of sterol-dependent membrane scaffolding may be critical for maintaining the correct subcellular localization, structural integrity or activity of the cellulose synthase machinery. As reviewed by Turner and Kumar [25], there is little evidence that steryl glucosides can act as primers for the attachment of glucose monomers during the synthesis of β−(1→4) glucan chains that form the cellulose microfibrils, while the most probable role of sterols has been proved in the process of membrane partitioning into sterol-rich microdomains in the region of cellulose synthesis. Sterols are also related to the membrane fluidity, thickness, and stability through interactions with membrane proteins and fatty acids [26]. In cauliflower meristematic tissues, sterols showed a role in water passage [21]. Femenia et al. [27,28] found that the cell wall of cauliflower florets, upper, middle, and lower stems undergo thickening during plant overripening through secondary wall formation (lignification) of vascular and supporting structures and decreasing in degree of methyl-esterification of parenchymatic cell walls, all causing tissue toughening on a macroscopic scale as well as strengthening of the cell wall, which entraps the intracellular phytochemicals on a microscopic scale. As reviewed by Jarvis [29], the mechanical properties of the cell wall cannot be deduced directly from the properties of the polysaccharides comprising them but from how they are interlinked to form the three-dimensional structure of the intact cell wall. There is also variation in cell wall structure at tissue, organ, and variety levels. Interlinking of cell-wall polymers by covalent, ionic, and hydrogen bonding varies and affects the strengthens of the cell wall and their compartmentalizing ability against the intracellular macromolecules.

Starting from this premise, cell wall structure in both the fresh and cooked vegetable tissues must be considered to have key dietary properties in terms of bioaccessibility of those intracellular phytochemicals having relatively high molecular size. The extent of pectin solubilization as induced by heat may lead to an erosion of the polymer assemblies in the cell wall microdomain, and therefore may reduce tissue compartmentalization on a macroscopic scale, thus enhancing the release of intracellular macromolecules as well as the access of enzymes such as α-amylases during the digestion process.

The present study was the first attempt to unravel the role of the main polymer assemblies at a cell wall scale in texture softening, tissue decompartmentalization kinetics, and changes of the extractability of sterols and tocopherols under heating. The effects of three cooking conditions, i.e., boiling, steaming, and *sous-vide*, on two varieties (*Depurple* and *Cheddar)* of cauliflower were investigated. The effectiveness of cell wall microstructure to undergo loosening was parameterized in a tailored rheological model, thus providing a powerful tool to maximize the extractability of the total amount of sterols and tocopherol. Changes, as induced by heat, in cauliflower microstructure as well as in their rheological properties may represent the keystones for design, optimization, and assessment of the cooking treatments when the aim is the maximizing of the nutritional value linked to the increase of bioaccessibility to the intracellular macromolecules having functional properties, by keeping texture acceptable for the consumption.

## 2. Materials and Methods

**Scheme 1:**
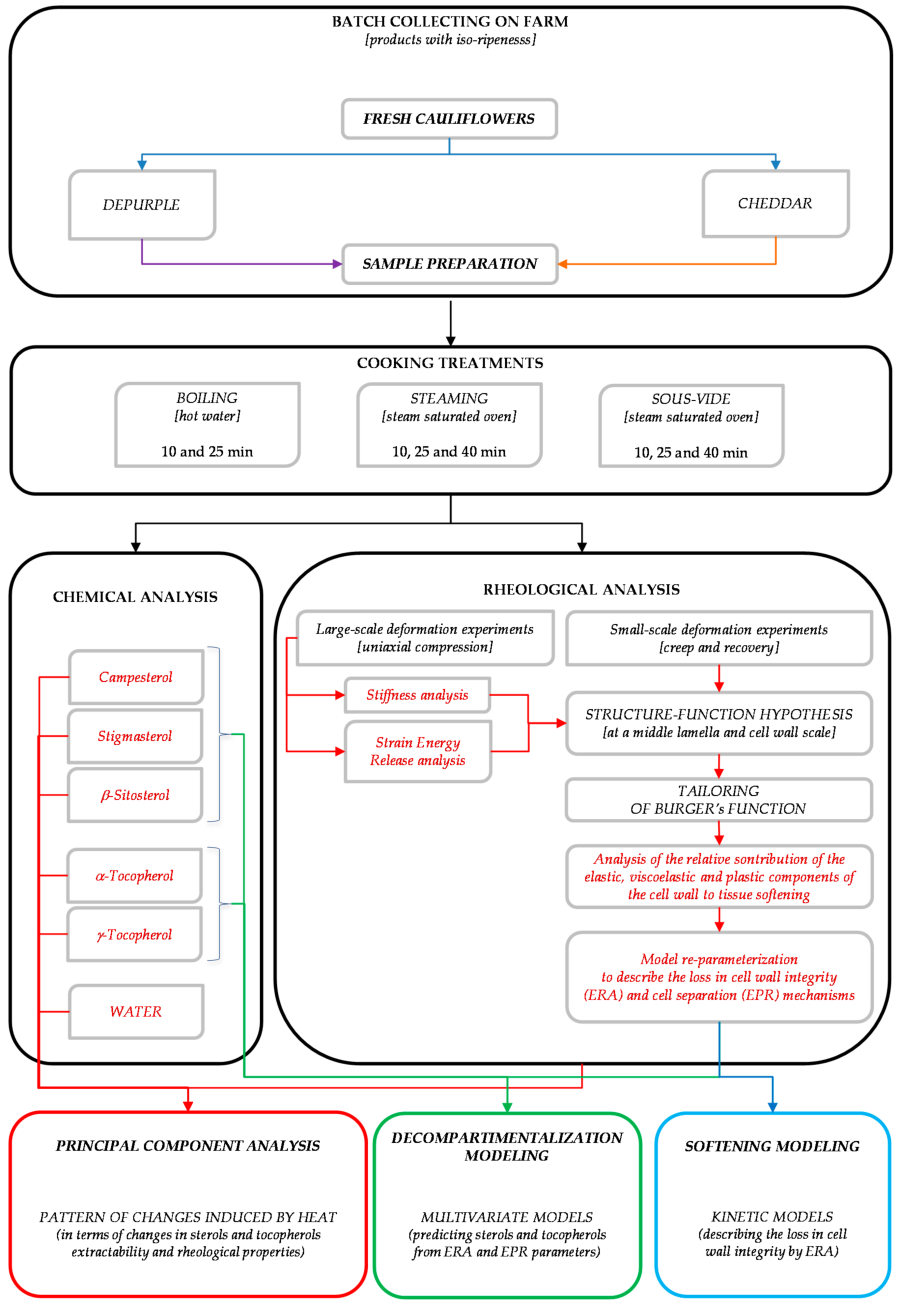
Flow diagram that explains the experimental methodology.

The experimental strategy is reported in general terms in Scheme 1.

### 2.1. Chemicals and Reagents

Tocopherols standards: α-tocopherol (α-T), β-tocopherol (b-T), γ-tocopherol, and δ-tocopherol), (>95% purity); phytosterols standards: 5α-cholestane, stigmasterol, β-sitosterol, and campesterol, >99%; solvents HPLC grade (>95% purity) such as acetone, acetonitrile, dichloromethane, ethanol, methanol, n-hexane, isopropanol, acetic acid, water, and reagents such as ascorbic acid (>99.5%), BSTFA (N,O-Bis(trimethylsilyl)trifluoroacetamide with 1% trimethylchlorosilane), and Supelclean cartridge LC-Si SPE (500 mg, 6 mL) from Merck (Darmstadt, Germany). Potassium hydroxide (85%) and anhydrous sodium sulfate from ITW Company (Darmstadt, Germany) were used as received. MilliQ water was purified with Millipore System (Millford, CT, USA).

### 2.2. Sampling

Six kg of colored cauliflower (*Brassica oleracea* L. var. botrytis), *Cheddar* (orange) and *Depurple* (purple) varieties were collected on November 2019 at the company Agrinovana S.r.l (Petritoli, Fermo, Italy). The two varieties underwent the same period of ripening before collecting.

Rosettes were removed of damaged tissue, leaves and stems, and was water washed and cut into small pieces of 9 ± 3 g of 3–4 cm diameter and 4 cm length. Then, they were selected by shape and divided into groups of 27 samples with a weight of 180–200 g each and finally destined to cooking tests.

### 2.3. Cooking Treatments

For boiling (B), one portion of cauliflower (180–200 g) was dipped in unsalted water (1.5 L) at boiling point in a pot of 18 cm diameter and cooked for 10 and 25 min. For steaming (S), a commercial oven (Bosch, HSG636ES1, MediaWorld, Italy) equipped with steam injection system was used to cook the cauliflower samples under full steam conditions (RH% = 100) at a nominal temperature of 95 °C (±0.5 °C) for three times of exposure (10, 25, 40 min). For *sous-vide* (SV) treatment, cauliflower was vacuum-packed in polypropylene heat-resistant (up to 120 °C) bag and submitted to full steam conditions as reported for S. In S and SV, the oven was not preheated before treatments. Each cooking trial was conducted in triplicate. Once cooked, cauliflower was equilibrated at room temperature. Thereafter, 54 samples of about 50 g were immediately destined to the rheological tests (9 cooking treatments and 6 replicates); the remaining 27 samples of about 130–150 g were knife-cut in small pieces, freeze-dried (Virtis Wizard 2.0 instrument, SP Industries, New York, NY, USA), homogenized and stored in vacuum pouches at −18 °C before to be destined to chemical analyses.

Time of treatment and nominal temperature in the three cooking methods were chosen aiming to simulate real household conditions when adopting traditional water boiling or modern commercial ovens as programmed for mild treatments (direct full steam oven and sous-vide).

Real-time time-temperature data were gathered by using a 34972A data acquisition/datalogger (Keysight Technologies, Milano, Italy) equipped with thermocouples of type K with time of response of 0.2 s and reproducibility 0.05 °C. Data were registered every 10 s during heating and cooling stages. Keysight PathWave BenchVue software allowed test configuration setup and real-time data display and analysis.

### 2.4. Water Content Determination

Raw and cooked samples were submitted to lyophilization process to extract the freezable water using a benchtop freeze drier (Virtis Wizard 2.0 instrument, SP Industries, New York, NY, USA). Freeze drying cycle included a cooling step (temperature was lowered from 23 °C to −40 °C in 1 h), a tempering step (temperature was kept at −40 °C for 4 h), a primary drying step (frozen water was sublimated under 540 torr) at −40 °C, and a second drying step (frozen water was sublimated under 540 torr at 23 °C). Temperature accuracy was ±0.1 °C.

The weight of samples was registered before and after lyophilization by using an analytical balance (Gibertini E42, Steroglass, Peurgia, Italy) and then compared to calculate the percent of extracted water. Weight accuracy was ±10^−5^ g.

### 2.5. Tocopherols Determination

Preliminary tests were conducted to optimize tocopherol determination by direct acetone extraction and saponification as reported by Nartea et al. [19] (See Figure S1 in Supplementary Material). A modified saponification procedure was optimized for cauliflower: 0.4 g of powder freeze dried vegetable was added of ascorbic acid (1 g), sodium sulphate (0.1 g), ethanol (20 mL), potassium hydroxide solution 80% (4 mL), and saponified in water bath (85 °C, 30 min), shaking from time to time. The sample was cooled at room temperature, added of water (12 mL), extracted three times with n-hexane (20, 10 and 20 mL), and centrifuged for better separation (2 min, 3600 rpm). The organic phases were pooled, washed four times with water (10 mL), dried with rotavapor at 35 °C and dissolved in n-hexane (1 mL), and split into two fractions: 10 μL was used for phytosterol determination (2.6) and 990 μL for tocopherol determination. Tocopherol analysis was run on a Waters Ultra Pressure Liquid Chromatographic Acquity system (UPLC Acquity H-Class, Waters Corporation, Milford, CT, USA) equipped with a Fluorimetric Detector (FLD) and an Ascentis Express Hilic column (15 cm × 2.1 mm, 2.7 μm). An isocratic elution of n-hexane (95.5%), isopropanol (0.4%), and acetic acid (0.1%) at 0.3 mL/min was performed at 30 °C (column heater and sample loading). FLD was set with an excitation and emission wavelength of 290 and 330 nm, respectively (See Figures S2 and S3 in Supplementary Material). Tocopherols were identified by comparison of retention time with pure standards and quantified with external calibration. α-, γ-, δ-tocopherol calibration curves ranged from 3 to 100 μg/mL with R^2^ values higher than 0.986. Limit of detection (LOD) and quantification (LOQ) were as followed: α-tocopherol, 4 and 14, γ-tocopherol, 3 and 11 and δ-tocopherol, 2 and 7 ng/mL. β-tocopherol was not identified in samples, thus LOD and LOQ are not reported.

### 2.6. Sterols Determination

Freeze dried cauliflower was submitted to alkaline saponification (85 °C, 30 min) as reported for tocopherol determination [19]. The alkaline saponification allows to determine the esterified bounded sterols to the matrix. Glycosylated and acylated sterols require a prior acid hydrolysis, but in this study, they were not determined. A volume of 10 μL of the n-hexane solution was added with 10 μL of 5α-cholestane standard (1 mg/mL in n-toluene), taken to dryness under nitrogen flow, added with BSTFA (N,O-Bis(trimethylsilyl)trifluoroacetamide with 1% trimethylchlorosilane) allowing the derivation reaction for 15 min at room temperature. The sample was taken to dryness and dissolved in 250 μL of n-hexane. All samples were injected (1 μL) in a GC/EI-MS (Thermo Scientific, Waltham, MA, USA) system, equipped with a split/splitless injector, and single quadrupole analytical column Rtx-65TG (30 m × 0.25 mm ID, 0.1 μm dF. Da). Oven temperature was set at 200 °C, held for 1 min, increased to 280 °C (2 °C/min) and held 1 min, using helium flow at 1.2 mL/min. The injector in splitless mode was set at 320 °C, the ionization source (70 eV) was set at 250 °C and auxiliary line at 280 °C. The acquisition was performed in total ion current (TIC) in a mass range of 70–650 *m*/*z* and with a detector gain of 1.0. Trimethylsilane-phytosterols were identified by comparison with pure standards of each phytosterol (retention time and mass spectra) (See Figures S4 and S5 in Supplementary Material). Quantification was performed by internal calibration using 5α-cholestane standard. LOQ was 1.8 mg/L.

### 2.7. Instrumental Evaluation of Texture Softening

Texture softening as induced by heat was investigated in the range of high-scale of deformation by analyzing the mode of failure under uniaxial compression as well as the in the range of small-scale of deformation by analyzing the load-bearing ability under creep (loading) and recovery (unloading) conditions.

Concerning the uniaxial compression tests, an amount of about 50 g fresh (unprocessed) and cooked cauliflower florets were placed on the stationary steel plate of a Universal Testing Machine (Zwick GmbH and Co, Ulm, Germany) equipped with a 2.5 kN load cell, with the convex side of the sample facing up and compressed with an upper steel plate. A preload of 5 N was reached with a crosshead speed of 5 mm/s and 10 s of resting time to relax preload stress; then, compression tests were conducted under deformation-controlled mode with a crosshead speed of 10 mm/s and loading was stopped at 50% deformation.

Creep and recovery tests were conducted under small-deformation conditions. A creep load target of 100 N was reached during loading step by imposing high crosshead speed (600 N/min) and kept for 600 s (10 min), and then the target load was removed, and the displacement was informed for 600 s (10 min) during the unloading step, allowing the equilibrium recovery in the residual structure.

All rheological tests were performed with 6 replicates.

### 2.8. Statistical Analysis

Sterols and tocopherols concentration data were reported as mean values ± standard deviation (SD) of three replicates. Data were analyzed by ANOVA and Tukey’s mean comparison test at a significance level of *p* < 0.05.

Creep behavior and decompartmentalization kinetics were analyzed by non-linear regression analysis of two mathematical models using Robust algorithm and Profit software ver. 7 (QuantumSoft, Zurich, Switzerland). The goodness of fit was evaluated by calculating the mean absolute relative error. Model parameters were estimated together with their 95% confidence intervals by performing 500 Monte Carlo simulations simultaneously.

Principal component analysis (PCA) was performed to highlight the pattern of changes induced by heat in terms of extractability of sterols and tocopherols as well as of microstructural-related properties.

Multiple regression analysis (MRA) was conducted to develop predictive models for the concentration on dry basis of total sterols, tocopherols, and water from cauliflower. The MRA models were developed and cross-validated on independent datasets. The original dataset related was divided in “calibration dataset” (including 70% of the samples of the original dataset) from which the MRA models were derived and “validation dataset” (including 30% of the samples of the original dataset) against which the MRA models were validated. The predictive accuracy of the MRA models was evaluated by calculating the mean absolute relative error as well as the R^2^ of the linear regression of predicted vs. observed with a statistical significance of *p* < 0.05. Coefficients of the MRA models were obtained using stepwise method together; their standard errors were also calculated from the inverse and transpose matrix of partial derivatives according to Brown (2001).

All statistical analyses were conducted using Statistica 10.0 (StatSoft 2011; Tulsa, OK, USA) except for ANOVA and Tukey’s test, which were performed using R software (version 3.5.0, The R Foundation for Statistical Computing).

## 3. Results and Discussion

The effects of the heat in both cauliflower varieties, *Cheddar* and *Depurple,* were evaluated under boiling (B), steaming (S) and *sous-vide* (SV) in terms of texture softening, tissue decompartmentalization kinetics and sterols and tocopherols extractability, using large- and small-scale deformations properties as structure-related descriptors on a macroscopic and microscopic scale, respectively.

### 3.1. Load Bearing Ability in the Range of Large-Scale Deformation

**Figure 1:**
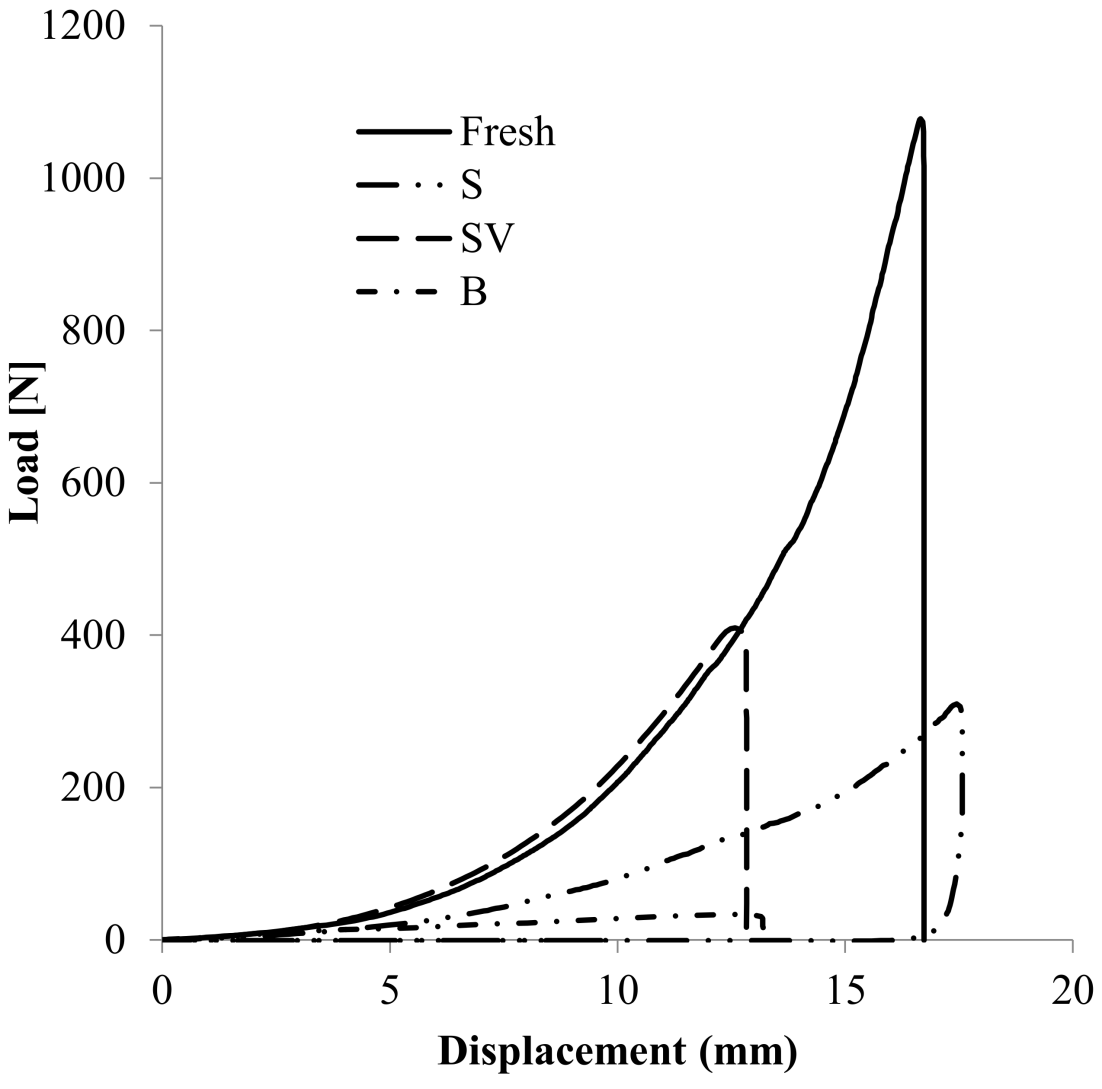
Load-bearing ability in the range of large-scale deformation of untreated (Fresh) and 25-min heated cauliflower (*Cheddar*) under steaming (S), *sous-vide* (SV), and boiling (B).

Load bearing ability was first evaluated by analyzing the relationships between load and displacement in the range of pre-failure deformation. Texture softening induced by the heat was examined by comparing the changes observed in the compression curves corresponding to the cooked samples with respect to the fresh (untreated) ones. Figure 1 shows the compression curves corresponding to the fresh and 25-min heated *Cheddar* cauliflower under boiling (**B**), steaming (**S**) and *sous-vide* (**SV**).

Similar trends were observed after 10 min of heating, while no meaningful load-displacement signals were registered after 40 min of heating under boiling due to the extreme texture softening (data not showed). Previous researchers [30] obtained similar load-displacement curves in studies focused on the effects of in-tap water boiling (95 °C) on cauliflower firmness. As can be inferred from Figure 1, no yield point was detected. The curves showed an upward concavity over the full-range of the experienced deformation (0.00–0.50). Preliminary tests based on cyclic loading-unloading steps (data not showed) confirmed that a reversible stress contribute must be considered as a result of the increase of the cauliflower surface in contact with the instrumental plate during the early step of compression (<0.15). However, the mechanical behavior observed in the successive steps of compression (>0.15) suggested an elastoplastic structure of cauliflower organs that was characterized by strain-hardening capacity, as suggested by the increasing in load bearing ability with increasing deformation. Area under load-displacement curves (referred as to “work-to-deformation”) as well as the degree of concavity in the load-displacement curve (referred as to “strain-hardening capacity”) were considered the result of simultaneous mechanisms occurring under mechanical deformation: (i) increase of resistance (stress) of the elastic and viscoelastic elements present in the cell wall, all counterbalancing the increase of turgor pressure, and (ii) new formation of cross links and entanglements in the middle lamella, causing loss in porosity, increase of cell-to-cell cohesivity and tissue toughening. As reviewed by Cosgrove [31], resistance to deformation due to a pre-stress in the plane of the cell wall as well as the resistance stemming from outward out-of-plane force of turgor pressure when the wall is sufficiently pressed into the cell can be considered potential factors explaining the non-linear stress-strain behavior and strain hardening response in plant cells under uniaxial deformation.

Texture softening induced by heat on a macroscopic scale was also evaluated using stiffness and strain energy release as two structure-related descriptors. stiffness (N/mm) measures the resistance to the deformation and was calculated as ratio between the load (N) corresponding to 0.5-level deformation (reached at the maximum displacement) and the maximum displacement (mm); then, it was used as a descriptor of the strain-hardening capacity. Strain energy release (N∙mm) was calculated as numerical integration of load, displacement data from preload to 0.5-level deformation, and then it was used as descriptor of the work-to-deformation. Decreases in strain energy release as induced by heat was associated with the cumulative damage caused in cell membrane (loss of turgor), cell wall (cell opening), and middle lamella (cell-to-cell separation). Decreasing in stiffness as induced by heat was associated with the damage caused in the cell membrane (loss of turgor) and middle lamella, mainly due to the pectin degradation, causing cell separation. As reviewed by Jarvis et al. [29], thermally induced β-elimination reaction, which occurs during the cooking of vegetables, is a relatively specific method of depolymerizing pectic galacturonans esterified on the galacturonoyl carboxyl group with no known effect on other covalent bonds within the plant cell wall. During cooking, it is commonly accompanied by chelation of divalent cations by organic acids released from within the cell, but it is not normally sufficient to induce cell separation after the cells are dead and the mechanical stress induced by turgor pressure is partly or completely loss as induced by heat.

**Figure 2:**
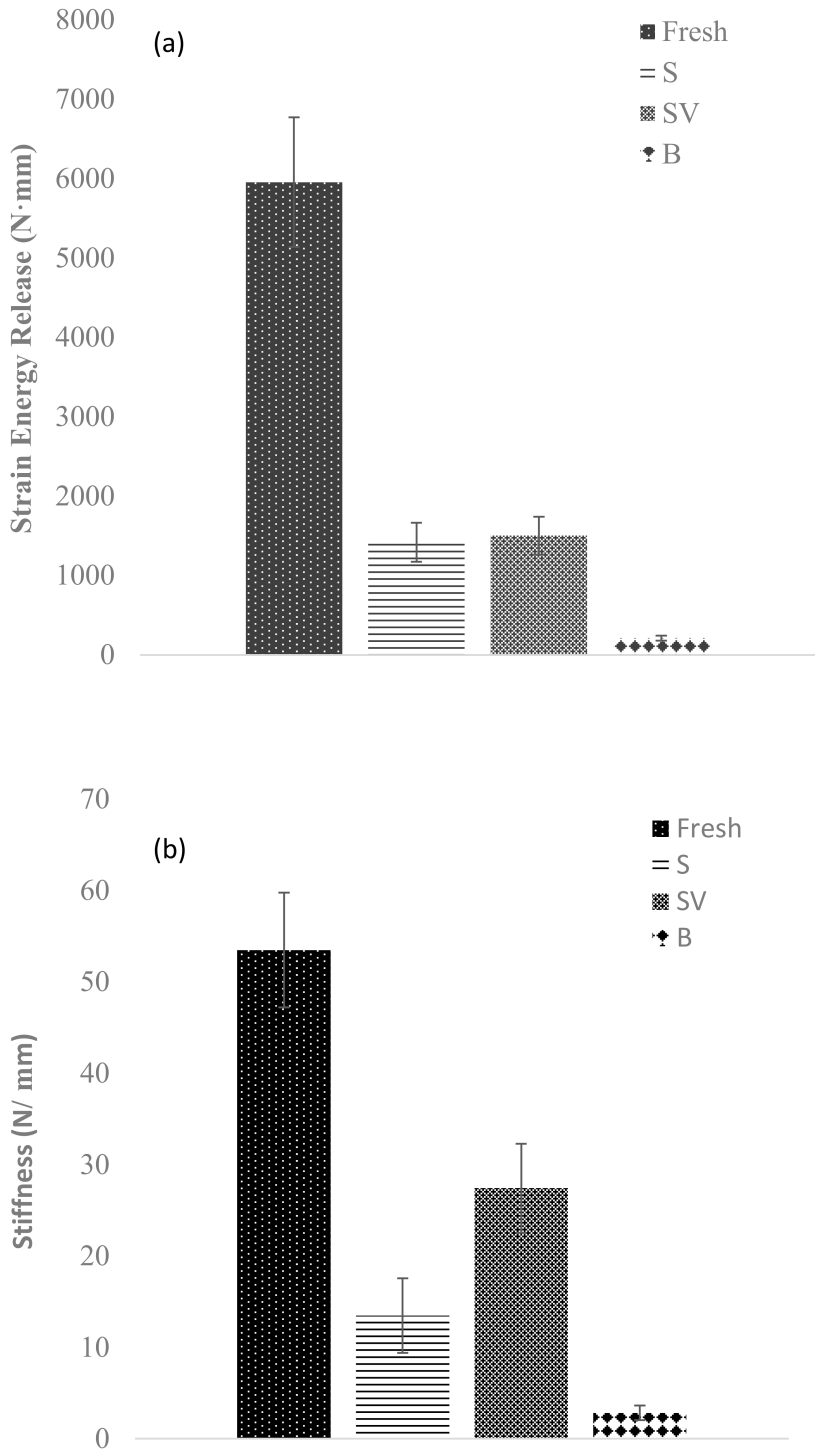
Strain energy release (**a**) and stiffness (**b**) levels corresponding to the untreated (Fresh) and 25-min heated *Cheddar* samples under steaming (S), *sous-vide* (SV), and boiling (B). Error bars were calculated from six compression curves.

Figure 2 shows the strain energy release and stiffness levels corresponding to the fresh and 25 min heated *Cheddar* samples under B, S, and SV conditions.

According to the strain energy release parameter, 25 min of heating under S and SV caused an “equivalent thermal effect” (differences were not significant, *p* < 0.05). However, the residual texture after S and SV was significantly lower (about 75%) than the initial level (fresh samples) and significantly higher (about 85%) than after boiling. Furthermore, 25 min of heating under boiling caused the loss of about 92% of the mechanical resistance at 0.5 deformation as compared to the fresh cauliflower. According to the stiffness parameter, equivalent thermal effects were not observed among cooking treatments: *sous-vide* steamed samples showed the highest residual stiffness among the cooked samples, while direct steamed samples showed an intermediate level between sous-vide steamed and boiled ones. Conversely, cauliflower boiled in water was characterized by the lowest level in the strain energy release and stiffness parameters among the cooked samples.

Because direct steamed and *sous-vide* steamed samples were characterized by an equivalent strain energy release (intermediate between boiled and untreated samples) and at the same time by different stiffness levels, it is suggested that the different cooking methods may induce the same extent of softening on a macroscopic scale through two independent disassembling mechanisms that occur on a microscopic scale, i.e., cell wall separation and cell wall rupture. Changes in rheological properties in the range of large-scale deformation, reported in Figure 1 and Figure 2, were also compared to the corresponding temperature profiles experienced by the samples under heating.

As referred in Jarvis [29], as soon as the integrity of the cell membrane is lost during heating, solutes of low-molecular weight can be released from the cells; however, the pore size of native cell walls is small enough to prevent the exit of macromolecules above about 10–20 kD and inhibit the access of digestive enzymes (e.g., lipases and amylases) that are larger than this size. Fractured cells will release their contents, and cells that remain intact may also do so if the pore diameter of their cell walls increases, for example due to pectin degradation.

Since the question of whether cell walls lose (rupture) or separate as induced by heat is relevant to the softening rate and location of intracellular macromolecules decompartmentalization. The effectiveness of the cell-wall microstructure in resisting these two kinds of disassembling mechanisms during cooking was evaluated in this work by analyzing the rheological properties on a cell scale size.

### 3.2. Modeling Creep Compliance at a Cellular Scale

Due to their intrinsic low sensitivity in load and deformation, large-scale deformation data cannot be suitable for assessment of the contribute of the basic elements of tissue microstructure, which include cellulose, hemicellulose, and pectin assemblies, to the tissue decompartmentalization as caused by the heat. As reviewed by Cosgrove [31], due to the wall anisotropic biosynthesis in growing cells and to the complexity of microstructure at a local scale, wall mechanics will necessitate recognition of at least three distinct components of the volumetric elastic moduli as measurable along with the three orthogonal directions of length, with and thickness of a cell strip under micro-indentation experiments. For this reason, creep and recovery test were conducted in the range of small-scale deformation.

**Figure 3:**
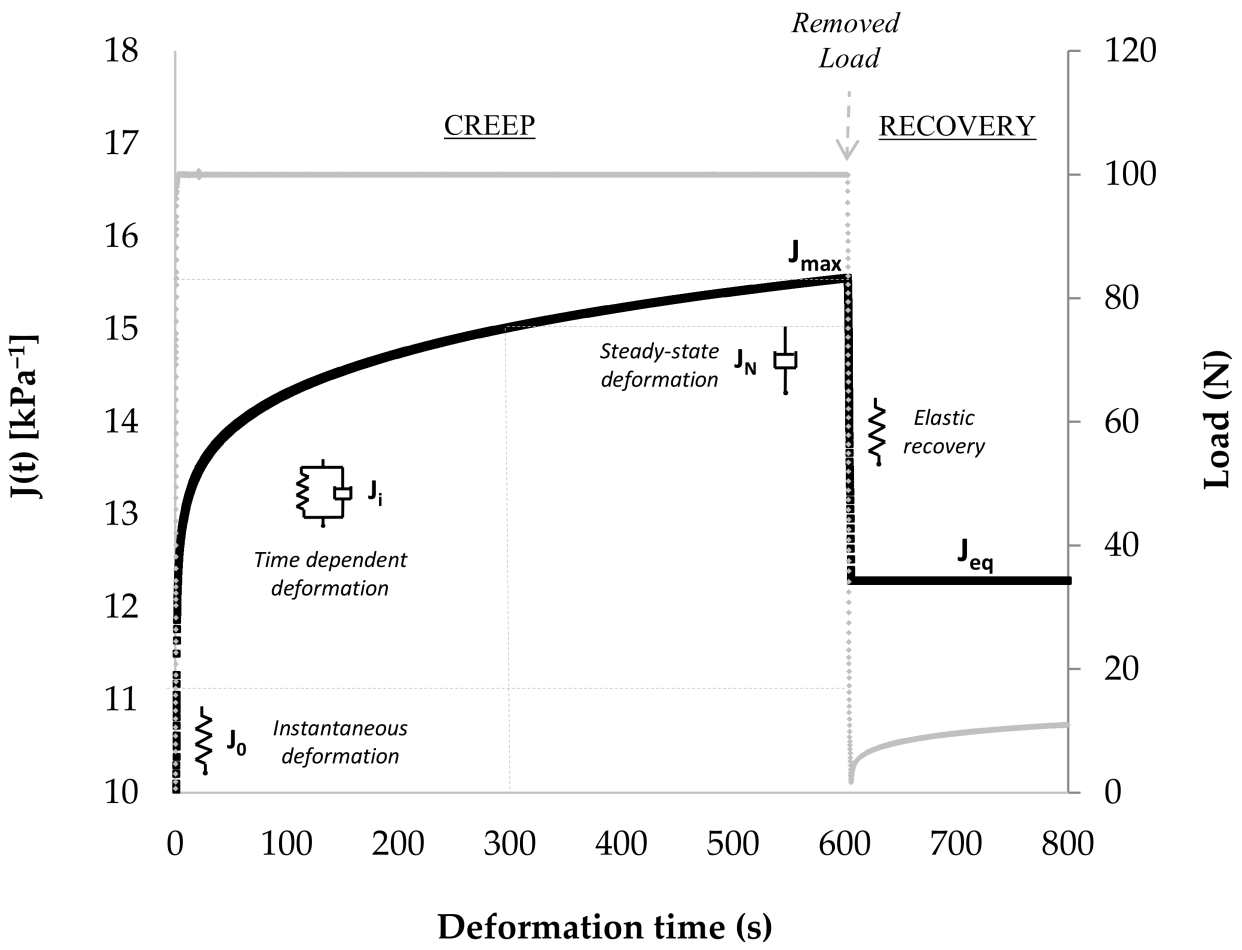
Creep compliance and recovery profile of *Cheddar* cauliflower after 25 min of heating under steaming.

Figure 3 shows the time-dependent compliance, namely J(t), registered during creep and recovery test of the *Cheddar* cauliflower after 25 min of steaming.

The curve was obtained by converting of displacement (mm) into compliance (kPa^−1^) data: J(t) was considered as the reciprocal of the uniaxial component of the volumetric elastic modulus (kPa) or, equivalently, the ratio between deformation (adimensional) and applied constant stress (kPa). As can be argued from the figure, changes in creep compliance registered during the loading step taken place through three distinguishable rheological responses. Quasi-instantaneous increasing compliance (J_0_) is followed by retarded relaxation (J_i_) occurring for about 200–300 s, increasing toward steady-state regime (J_N_) before to reach the maximum level (J_max_). The equilibrium deformation (J_eq_) as determined at the end of the recovery step was associated with the extent of irreversible damage caused by the applied load. The temporal succession of the compliance responses corroborated the idea that the cauliflower tissues are characterized by an elastoplastic structure at cell scale and that elastic, viscoelastic and viscoplastic relaxation mechanisms are involved to determine their compliance under external loading at different levels of the structure hierarchy. This means that the cell structural elements interact hierarchically and additively, all contributing to counterbalance both the intracellular turgor pressure and external stress through a wide spectrum of relaxation mechanisms.

Based on stress relaxation and creep experiments, several studies have shown that mechanical properties of the plant tissues at a macroscopic scale originate from the cell wall hierarchical organization both at nano- and microstructural scales [32,33,34]. As reviewed by Cosgrove [31], cellulose microfibril is the main elastic load-bearing component, while pectin plays a plastic role decreasing modulus and modifying extensibility of cellulose fibril assemblies. Again, the presence of pectin polymers in the cellulose matrix reduces its elastic properties in proportion of their content, while xyloglucans incorporation in cellulose matrix or in a cellulose-pectin matrix leads to a compliant material characterized by time-dependent (viscoelastic) creep behavior.

In this work, the overall creep compliance profile registered in the range of small-scale deformation before and after cooking was considered as a fingerprinting of the load-bearing ability of the cauliflower tissue microstructure, to which the basic elements of the cell wall contribute hierarchically and additively through elastic, viscoelastic and viscoplastic relaxation mechanisms. With the aim to evaluate the effectiveness of the main cell components in resisting cell-to-cell separation and cell wall loosening mechanisms as affected by heating, an elastoplastic mechanical model was proposed to describe quantitatively the creep behavior. Such a model was based on a hypothesis of structure-function relationships of the supramolecular assemblies among cellulose, hemicellulose and pectin and it was conceptualized by accounting for the manner that they appear interconnected in the cell wall and middle lamella microdomains as widely documented in literature. Excellent reviews and detailed papers have been published over the last two decades focused on polymer composition and hierarchical interconnection on a supramolecular scale in plant cell walls and middle lamella as well as on their mechanical properties. As reviewed by Cosgrove, Ranganathan et al., and Jarvis [1,29,31], the microstructure of the primary cell wall of vegetable tissues can be envisaged as four structurally independent but interacting polymeric domains. The first domain consists of parallel layers and lamellae of cellulose microfibril (linear chains with β-(1,4) covalent bonds among glucosyl residues) with crystalline and amorphous regions that are assembled in a second domain, a three-dimensional network of hemicellulose (consisting of xyloglucans and acidic arabinoxylans). Hemicelluloses are linked to cellulose microfibrils through hydrogen bonding, with evidence for covalent linkage for arabinoxylans to cellulose through diferuloylesters that plays a role in microfibril spacing and that provides shape and strength to the cell wall. Cellulose-hemicellulose network is embedded in a third domain, the middle lamella, consisting of water-soluble calcium-binding pectin (about 30% of the dry matter of the primary cell wall), which can be considered as an extension of the previous domains from which the cellulose microfibril and calcium are lacking. Pectin is a hydrated gel-type matrix of heteropolysaccharides rich in D-galacturonic acid, including neutral sidechains of polysaccharides, consisting of linear homogalacturonan and branched blocks of rhamnogalacturonans. The cementing function of pectic substances among the individual cells depends mainly on their non-methyl esterified homogalacturonan regions, which form “egg-box” structures through ionic binding with bivalent ions (mainly calcium). There is evidence that pectin may be also covalently cross-linked through covalent bonds to phenolic acids and hemicelluloses by means of ferulic acid dimers. The fourth domain of cell wall structure consists of structural glycoproteins, oriented radially within the primary cell wall network, of which are water soluble and with lubrication (viscous and plastic) properties.

**Scheme 2:**
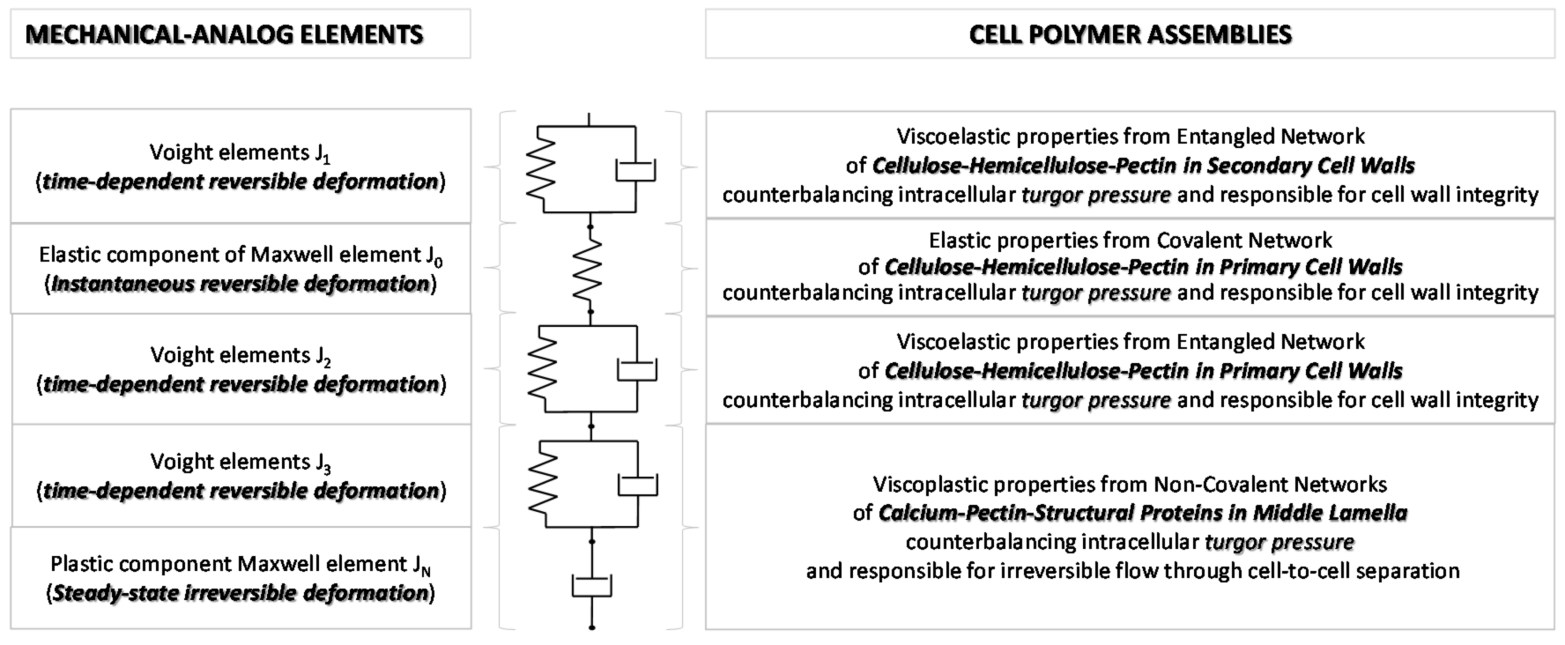
Tailored Burger’s array illustrating the hypothesis structure-function relationships among the polymeric assemblies at cell wall and middle lamella microdomains.

Scheme 2 illustrates the hypothesis relationships between mechanical analog elements in the proposed model, pictured as a tailored Burger’s array, and cell polymer assemblies at cell wall and middle lamella microdomains.

Each mechanical element in the model was here associated with specific supramolecular assembly of cellulose, hemicellulose and pectin interacting additively at distinct levels of the microstructure hierarchy. One in-series independent viscoelastic spring-dashpot arrays (Voigt element, **J_1_**), describing the first component of the time-dependent reversible compliance in the model, was associated with the entangled network assemblies of cellulose microfibril, hemicelluloses, and pectin located in the secondary cell wall, the young part of the cell wall. The lack of an elastic spring in such a microdomain was justified by the high proportion of meristematic cells in the cauliflower under investigation. One *in-series* independent spring (elastic component of the Maxwell element, **J_0_**), describing the reversible and instantaneous compliance in the model, was associated with the network of all covalent bonds (with elastic properties) in cellulose microfibril, hemicellulose and pectin located in the primary cell wall microdomain, i.e., the oldest part of the cell wall. Such a microstructural assembly is located at the intermediated level between the secondary wall and middle lamella, and it is considered as the principal cell element able to counterbalance the intracellular turgor pressure from plasma membrane by engaging mainly the cellulose backbone, which the heat cannot degrade. As reviewed by Cosgrove [31], cellulose microfibrils are deposited in the plane in the cell wall microdomain, thus it was expected to greatly influence the in-plane component of the volumetric elastic modulus. Two in-parallel interacting viscoelastic spring-dashpot arrays (Voigt elements, **J_2_**_,_ and **J_3_**), describing the second and third components of the time-dependent reversible compliance in the model, were associated with the entangled network assemblies of cellulose microfibril, hemicelluloses, and pectin located in the primary cell wall and middle lamella, respectively. The entangled network assemblies, as represented by the three spring-dashpot arrays, were cumulatively considered as the secondary cell element able to counterbalance turgor pressure by engaging simultaneously covalent bonds (with elastic properties) and cross-links (with time-dependent viscoelastic properties) that the heat can only partly degrade. One *in-series* independent dashpot (plastic component of the Maxwell element), describing the steady-state compliance regime in the model (namely **J_N_**), was associated with the gel network among calcium, pectin and structural proteins that spreads from the middle lamella microdomain to that of the primary cell wall, and that the heat can rapidly and completely degrade. As reviewed by Cosgrove [31], pectin assemblies probably exert dominant control of the out-of-plane component of volumetric elastic modulus, thus in this work, it was considered responsible for the viscoplastic (irreversible) flow occurring during through cell-to-cell separation, without decompartmentalization.

The overall compliance of cauliflower in the range of small-scale deformation was mathematically described using the following tailored Burger’s function:

(1) $$J\left(t\right){=J}_{0}{+J}_{i}\cdot {\left[1\;-\;\exp\left(-\frac{t}{{\mathrm{RT}}_{i}}\right)\right]}_{i}{+J}_{N}\;\cdot \;t$$

**J_0_** (kPa^−1^) is the instantaneous reversible compliance associated with the elastic stretching of the covalent bonds having the highest degree of freedom in the linear polymer fractions present in the cellulose microfibril network.

**J_i_** (kPa^−1^) is the retarded reversible compliance of the i^th^-viscoelastic elements in the cellulose-hemicellulose-pectin network (they are associated with the stretching of covalent cross-links, the rate of which can be slow down by the simultaneous disentanglements/disassembling of non-covalent bonds interacting each other).

**RT_i_** (s) are the characteristic relaxation times governing the retarded compliance under viscoelastic regime.

**J_N_** (kPa^−1^) is the steady-state irreversible compliance. It is associated with the rupture of non-covalent bonds (calcium-pectin gel) in the middle lamella and among the structural proteins of the cell wall, both enabling a viscoplastic flow through partial cell separation and cell wall rupture.

All six replicates of the experimental creep curves were used simultaneously as an input in the non-linear regression of the proposed elastoplastic model (Equation (1)) accounting for an increasing number of the viscoelastic elements, and Durbin-Watson parameter was used as criterion to retain in the model the three fitting terms, namely **J_1_**, **J_2_**, and **J_3_**, with the lowest degree of correlation among model residues with zero mean.

The average value of all Burger’s model parameters was estimated under all investigated heating conditions and the lack-of-fit of the model was evaluated by calculating the square relative mean error

(2) $$\tilde{E}\%=\frac{100}{N-3}\cdot \sqrt{\frac{{\left[J\left(t\right)-J{\left(t\right)}_{\exp}\right]}^{2}}{J\left(t\right)}}$$

The average values of the Burger’s model parameters are reported in Table 1. **E%** resulted to be always less than 2, suggesting that the structure-function relationships hypothesis was adequate to satisfactorily describe the overall creep behavior of cauliflower in the range of small-scale deformation before and after heating.

**Scheme 3:**
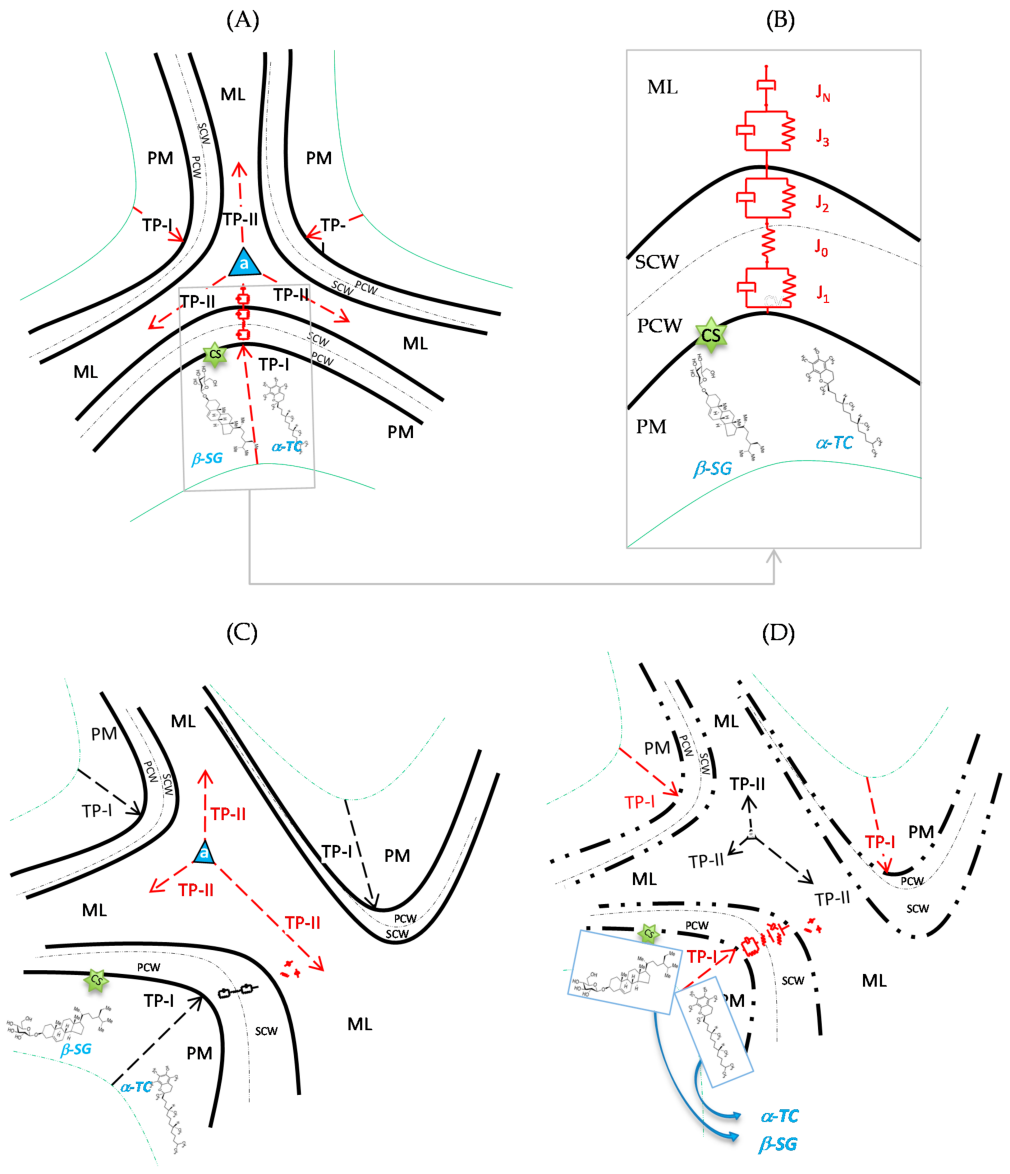
Biophysics underlying softening and decompartmentalization as induced by heat. At the equilibrium, the mechanical stress induced by cell turgor is distributed at tricellular junctions joining the parenchymatic cells with two stress components: the first component acting at cell wall level on the plane of each cell-to-cell contact, and the second component acting at middle lamella level as radial stress separating the cells at the tricellular joining corners (**A**). Polymer assemblies contribute to counter balance turgor by stressing effectively their elastic, viscoelastic and plastic elements from middle lamella to primary and secondary cell wall microdomains (**B**). Heating causes partial disassembling of plasma membrane (reducing turgor) and pectin degradation, mainly through enzymatic and chemical reactions. When pectin degradation is limited in the middle lamella microdomain, the survivor second stress component of turgor will be able to separate the cells without rupture of their walls, with sterols and tocopherols remaining entrapped by the integer walls (**C**). When pectin degradation extends from middle lamella to primary and secondary cell wall, the survivor first stress component of turgor will be able to cause wall loosening (or partial rupture) and effective decompartmentalization of sterols and tocopherols (**D**). TP-I, turgor pressure I component; TP-II, turgor pressure II component; PCW, primary cell wall; SCW, secondary cell wall; PM, plasma membrane; ML, middle lamella; CS, cellulose synthase; β-SG, glucoside of β-Sitosterol; α-TC, α-Tocopherol.

The main advantage of tailoring the generalized Burgers’ function was to evaluate in an independent way the effect of the heat on the cauliflower tissue in reducing the effectiveness of the individual elastic, viscoelastic and plastic components of the polymeric assemblies located in the cell wall and middle lamella microdomains to counterbalance the residual turgor pressure under stresses equilibrium. The role of the individual elastic, viscoelastic and plastic elements of the polymer assemblies in the cell wall and middle lamella microdomains underlying the biophysics of thermal softening on a macroscopic scale as well as cell-to-cell separation, cell wall loosening and phytochemical decompartmentalization on a microscopic scale is pictured in Scheme 3.

As reviewed by Jarvis et al. [35], pectin is the only polymer present in a tricellular junction joining the parenchymatic cells, and the mechanical stress induced by cell turgor is distributed at tricellular junctions with two components: the first stress component acting at cell wall level on the plane of each cell-to-cell contact, and the second stress component acting at middle lamella level as radial stress separating the cells at the tricellular joining corners. In this work, phospholipidic membrane was assumed as the most heat-sensitive cell element that can be immediately degraded by the heat, thus causing partial loss in turgor pressure and its two stress components in the early step of heating and complete loss of turgor after prolonged heating. The second stress component of the turgor pressure surviving on heating was considered responsible of the cell separation mechanism causing the increase in creep compliance during the steady-state deformation regime under creep (Figure 3). Cell separation on heating was associated with the degradation of pectin assemblies in the middle lamella microdomains. Otherwise, the first stress component of the turgor pressure that survives on heating was considered responsible of the increasing in creep compliance through partial rupture or loosening mechanisms of the cell wall and, therefore of the phytochemical decompartmentalization on a microscopic scale. Cell wall loosening or rupture on heating was associated with the degradation of the pectin assemblies in the cell wall microdomains, causing loss in the cell wall thickness or increasing of porosity, respectively.

Properties algebraically derived from the Burgers’ model parameters were proposed as descriptors of the overall creep compliance as well as at cell wall or middle lamella microdomains. The integrated compliance parameter (**U_r_** expressed as s/kPa) was calculated as numerical integral from zero to the time at load removing of the creep compliance curve and used to represents the energy releasing rate under creep. An increase of the **U_r_** parameter as induced by heat was treated as a quantitative measure of the cumulative softening of tissue microstructure across the two microdomains, i.e., cell wall and middle lamella. Since the compliance is an extensive property, the reversible compliance parameter, namely **J_R_**, was calculated as sum of the instantaneous J_0_ and retarded (**J_1_**, **J_2_**, and **J_3_**) compliances, and used to describe the cumulative changes as induced by heat in the three viscoelastic domains located in the primary and secondary cell wall and middle lamella. The ratios **J_0_**/**U_r_**, Σ**J_i_**/**U_r_** (with Σ**J_i_** calculated as sum of **J**, and **J_3_**) and **J_N_**/**U**_r_ were also calculated and used to compare the relative contributions from the elastic, viscoelastic and plastic components to the thermal softening.

**Figure 4:**
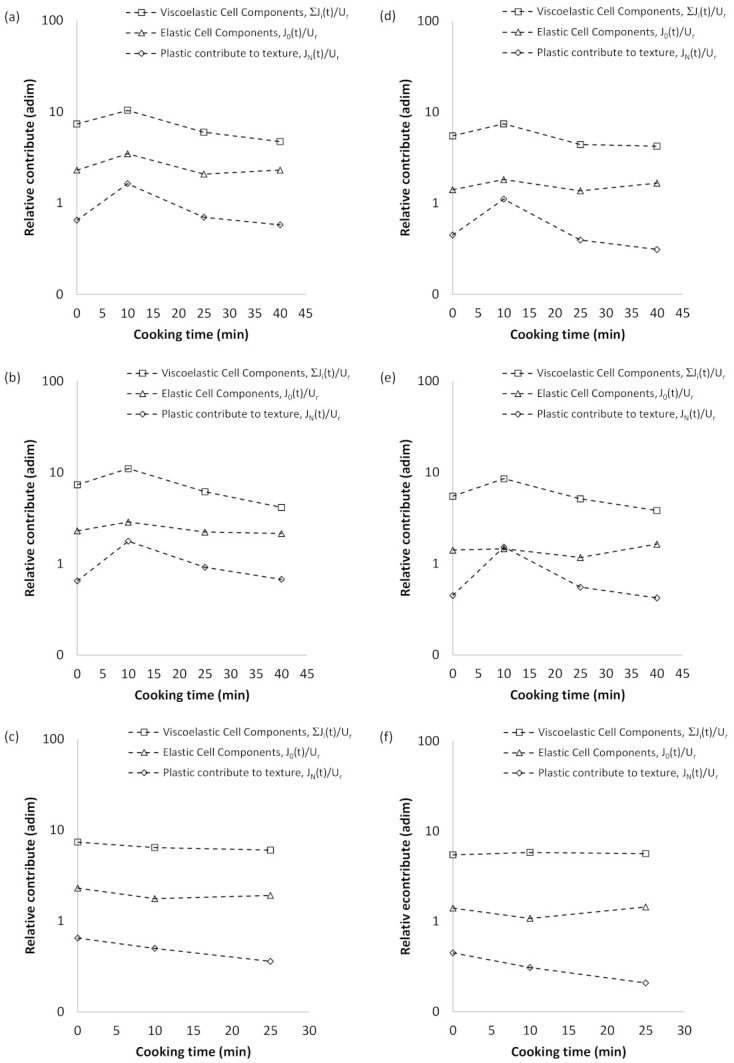
Relative contribution of the elastic, viscoelastic, and plastic cell elements to the overall creep compliance after steaming (**a**,**d**), *sous-vide* (**b**,**e**) and boiling (**c**,**f**) for *Depurple* (left) and *Cheddar* (right) cauliflower.

The relative contributions from the elastic **J_0_**/**U_r_**, viscoelastic Σ**J_i_**/**U_r_** and plastic **J_N_**/**U_r_** cell components to the tissue microstructure compliance were compared in Figure 4.

As it can be argued from figure, the three rheological parameters changes with the time of heating according to synchronous trends, suggesting that the two varieties of cauliflower are characterized by a tissue microstructure having the same microstructure hierarchy and polymer interactions at a cellular scale. Data also confirm that the relative contribution of cell elements to the overall creep compliance followed a decreasing order among viscoelastic, elastic and plastic supramolecular assemblies, either in the absence of the heat and after heating. As a consequence, the non-covalent network among cellulose, hemicellulose and pectin assemblies can be recognized as the most compliant and heat-sensitive cell element, irrespectively of the cauliflower variety and heating technology. In other words, the interconnections inside cellulose, hemicellulose and pectin assemblies represent the key scale level on which texture softening and tissue decompartmentalization are mainly depending on a microscopic scale.

**Figure 5:**
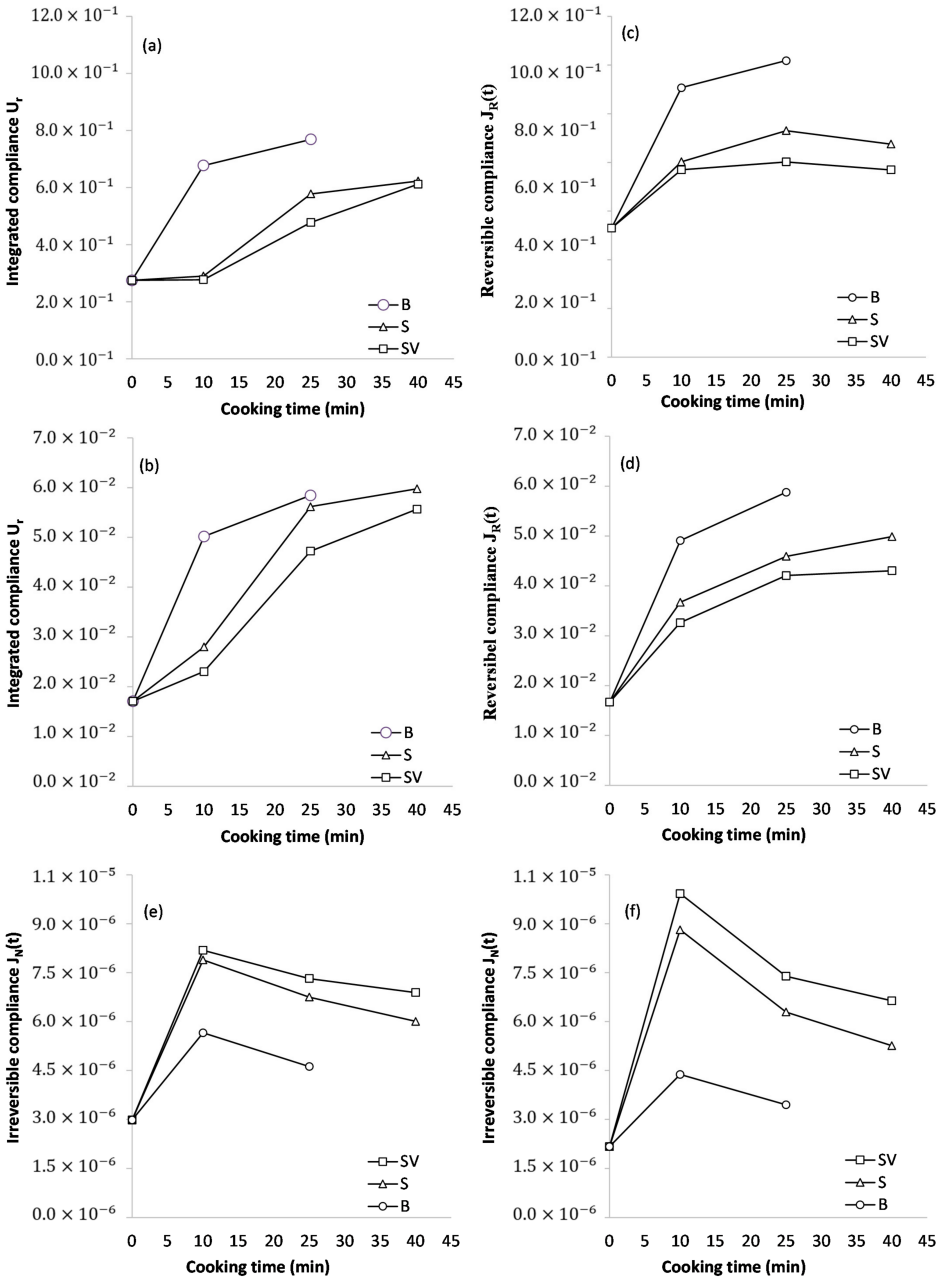
Softening kinetics as described by the integrated compliance **U_r_**(t) (**a**,**b**) as well as of the reversible **J_R_**(t) (**c**,**d**) and irreversible **J_N_**(t) (**e**,**f**) components of the compliance under boiling (B), steaming (S), and *sous-vide* (SV) for *Depurple* (left) and *Cheddar* (right) cauliflower.

Changes induced by heat in terms of the integrated compliance (**U_r_**) as well as of the reversible (**J_R_**) and irreversible (**J_N_**) components of the compliance are reported in Figure 5.

Overall softening of tissue microstructure as represented cumulatively by **U_r_** progressed with the time of heating according to a sigmoidal kinetics, while an asynchronous trend was observed between **J_R_** and **J_N_**. However, a common feature was observed during cooking among the rheological parameters. An apparent breakpoint was detected during the early step of heating (close to 10 min), easier to detect under boiling than steaming and *sous-vide*. The reversible compliance (**J_R_**) increased considerably (both the elastic springs and viscoelastic elements in the model were lost partially and promptly) toward an asymptotic level, whereas the irreversible compliance (**J_N_**) reached maximum level within 10 min of heating after which it decreased with the time of heating. Such a result was in agreement with literature. As reviewed by Ranganathan et al. [1] and more recently by Liu et al. [36], thermal softening in vegetables arises from two competing first-order mechanisms. The first mechanism was associated with chemical depolymerization of pectic substances in the middle lamella region via β-elimination mechanisms of their side chains, accounting for the loss of 85–97% of the original tissue firmness; the second mechanism was associated with the strengthening of the residual texture after prolonged heating through two independent firming mechanisms mediated by endogenous enzymes.

Under our experimental conditions, the synchronous increasing in **J_R_** and **J_N_** under creep after 10 min of heating was associated with two coupled relaxing mechanisms: (1) physical disruption of the bilayer phospholipidic membranes that cause partial loss in turgor, the surviving second stress component of which became less able to resist the external load, and (2) reorganization of viscoelastic and plastic elements in the middle lamella microdomain caused by water solubilization of calcium-pectic assemblies, thermal denaturation of structural proteins and chemical depolymerization via β-elimination of pectin side chains, all concurring locally to an increase of polymer mobility and to a decrease of cell-to-cell cohesiveness. Studies focused on carrots showed that many changes in pectin and loss in cellular turgor occur in early heating stages (namely within 3–10 min) when temperature reaches 100 °C and further changes are apparent after several minutes (30 min) when firmness is lost [37].

Under our experimental conditions, the asymptotic increase of **J_R_** occurring during the prolonged heating (from 10 to 40 min) was associated with the loosening (or partial rupture) of the viscoelastic elements extending from middle lamella microdomain (where the rate of the chemical depolymerization of pectin is slowing down by the enzymatic demethylesterification) to the primary and secondary cell wall microdomains, resulting in a decrease in cell wall porosity and thickness. Borowski et al. [38] provided evidence that parenchymatic cell wall of broccoli boiled in water undergo loosening and dissolution as indicated by the highest content of total pectin, protopectin and water-soluble pectin fractions in boiling water, while steamed samples undergo partial rupture of cell wall (with only 1–2% of dry matter loss). Similar results and hypotheses were provided by Christiaens et al. [39].

Under our experimental conditions, the decrease of **J_N_** from 10 to 40 min was associated with a partial strengthening of pectin assemblies in the middle lamella microdomain, more likely due to the enzymatic demethylesterification of pectin (mediated by endogenous pectinmethylesterases and polygalacturonases) followed by polymer reorganization through pectin-calcium binding. It is widely documented in literature that the endogenous pectinmethylesterase may act (i) randomly in de-methylesterification reactions of pectin side chains, thus promoting the action of endogenous polygalacturonase that results in the loss of firmness and decrease in susceptibility of pectin to further chemical degradation, and (ii) linearly, enhancing the formation of calcium bridges between free carbonyl groups of demethylesterified pectin chains forming pectate-calcium complexes, thus causing partial strengthening in the middle lamella. Changes in **J_N_** from 10 to 40 min of heating were more pronounced under steaming and *sous-vide* than under boiling, irrespectively of the cauliflower variety. Such a result suggested that boiling treatments conducted under our conditions probably were not effective to activate the endogenous pectinmethylesterases. As reported by Kapusta-Ducth et al. [15] in green and purple cauliflower, hydrothermal treatments cause consistent leakage of potassium and calcium in the hot water during the early phase of heating: this is another factor because the water boiling technology does not permit the consistent activation of pectinmethylesterases.

Under our experimental conditions, changes in **J_R_** were more pronounced under boiling, whereas changes in **J_N_** were more pronounced under steaming and *sous-vide*. This indicates that boiling was the most effective heating treatment to enhance loosening or rupture in the cell wall microdomain during early heating as well as that steaming and *sous-vide* provide the most of changes in the middle lamella microdomain. As reviewed by Baldwin [40], *sous-vide* cooking has shown to leave the plant cell wall mostly intact, and around 82–85 °C, it makes the vegetables tender by dissolving the cementing material that holds the cells together.

As far as the effect of heating on the two investigated varieties of cauliflower is concerned, although fresh samples showed no significant differences in terms of **U_r_**, the starting level of both **J_R_** and **J_N_** were in the *Cheddar* lower than in the *Depurple*. Furthermore, softening kinetics as described by **U_r_**, **J_R_**, and **J_N_** was faster and more pronounced in *Cheddar* variety than in the *Depurple* one and it progressed with a decreasing rate under boiling, steaming and *sous-vide*. Such differences were in close agreement with texture softening determined on a macroscopic scale through the large-scale deformation experiments. Differences in the starting levels of **J_R_** and **J_N_** as well as in softening kinetics between the two varieties of cauliflower were tentatively explained by supposing that the degree of pectin methylesterification in the middle lamella microdomain was in *Cheddar* cauliflower higher than in the *Depurple* one. Such a hypothesis was according to the asynchronous contributions from viscoelastic and plastic cell elements as described by the model parameters J_R_ and **J_N_**, respectively. In particular, untreated samples belonging to the *Depurple* variety were characterized by higher level of reversible compliance (**J_R_**). A higher number of methyl groups in the untreated samples will imply a greater proportion of hydrogen bonds and a higher creep compliance of both the viscoelastic (**J_R_**) and plastic (**J_N_**) cell wall elements. Additively, a higher number of methyl groups may accelerate the pectin depolymerization during heating, resulting in faster softening during the early step of heating (Figure 5b,d) as well as in faster strengthening through calcium-pectin binding during prolonged heating (Figure 5f). Our evidence-supported hypotheses concerning the contribution of the degree of pectin methylesterification in the mechanical properties as well as about the relationship between the softening rate and degree of methylation were in agreement with literature. As reviewed by Levesque-Tremblay et al. [41] changes in degree of methylesterification result in changes of cell wall elasticity in meristematic tissues. Fraeye et al. [42] provided evidence that high degree of methylesterification results in a more pronounced increase in the rate constant of chemical pectin depolymerization via β-elimination mechanisms rather than in the rate constant of enzymatic de-methylesterification. Fraeye et al. [43] reported that pectin with low degree of methylesterification promotes cation binding of pectin. Constenla and Lozano [44] showed that depolymerization of pectin in aqueous solutions causes an exponential reduction of the intrinsic viscosity (to one half after 40 min of heating at 80 °C) of the pectin polymer as a linear function of the degree of methylesterification.

Differences in softening kinetics (as described by **U_r_**, **J_R_**, and **J_N_**) among the investigated heating conditions were explained by supposing that pectin degradation undergoes chemical and enzymatic reactions with different rate and extent as a function of the actual time-temperature conditions experienced by the cauliflower during heating. In particular, the enzymatic degrading mechanism was supposed to be activated selectively according to the rate of increasing in temperature, reflecting the specific heat-transfer efficiency of the cooking methods. Steam condenses on the surface of the products and a large amount of latent heat transfers to the plant tissue with an intermediate rate between boiling and *sous-vide*.

It is largely accepted that, the optimal range of temperature for endogenous pectinmethylesterases typically is 50–80 °C, after which the enzymes are progressively inactivated in vegetables. Li Ni et al. [45] reported that pectinmethylesterases can be activated by heat activity in broccoli between 50 and 55 °C as well as from 50 to 70 °C in choy midribs; however, thermal stability of the pectinmethylesterases is lost during heating by following the first-order kinetics in the range of 70–95 °C. Pectinmethylesterases are completely inactivated in broccoli after 5 min treatment at 80 °C [46].

**Figure 6:**
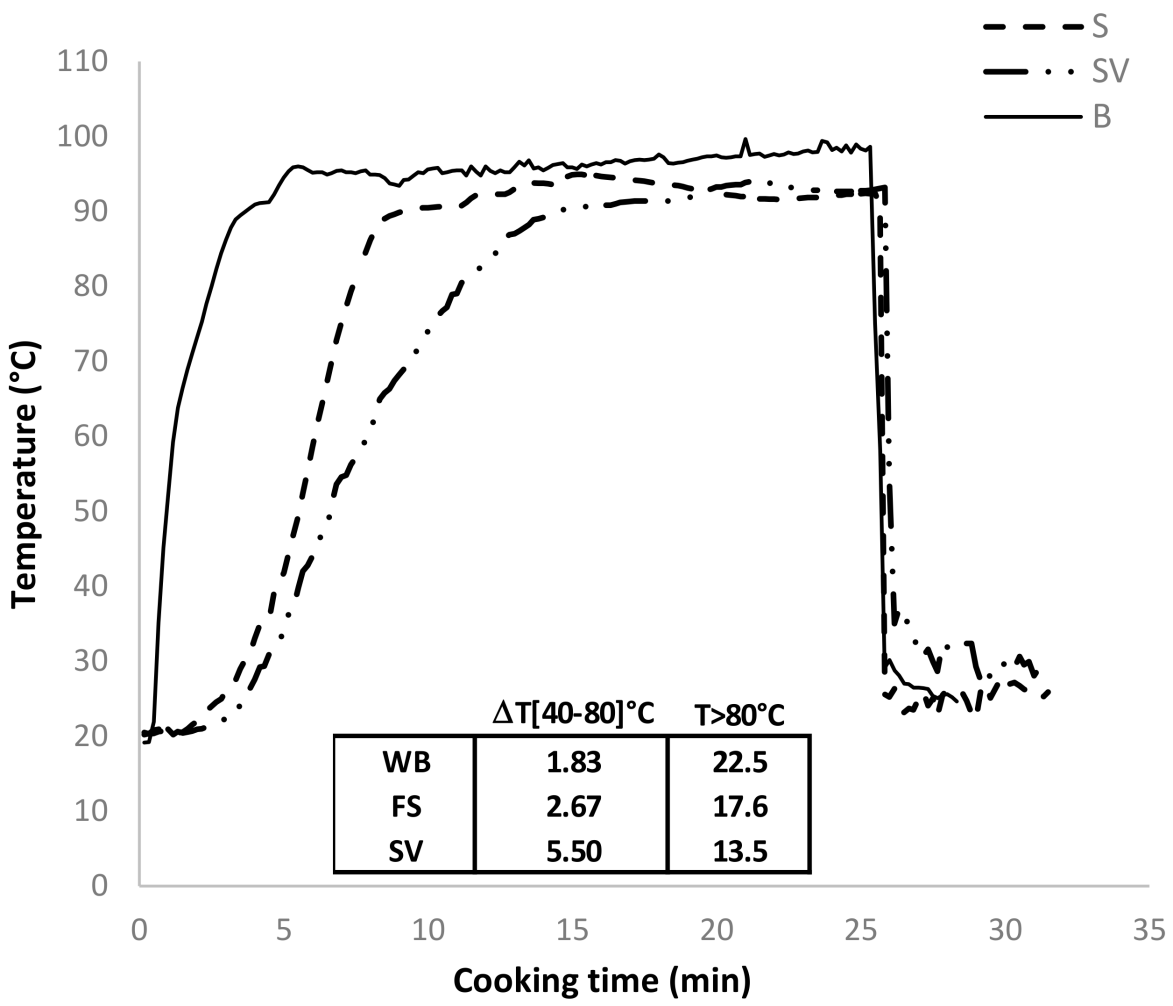
Time-temperature profiles under 25 min of steaming (S) and *sous-vide* (SV), and boiling (B), of Cheddar cauliflower. The inset shows the minutes of heating experienced from 40 to 80 °C (optimum range for pectinmethylesterases activity) and beyond 80 °C (deactivating of pectinmethylesterases).

Under our experimental conditions, the time of heating experienced by the samples in the temperature range potentially activating the endogenous pectinmethylesterases followed a decreasing order under boiling, steaming and *sous-vide*. For the sake of example, Figure 6 shows the time-temperature profiles of 25 min boiling, steaming and *sous-vide* treatments for *Cheddar* cauliflower. As can be inferred from data, samples experienced the increase in temperature from 40 to 80 °C in about 1.83, 2.67, and 5.5 min under boiling, steaming and *sous-vide* treatments, respectively. Furthermore, the time experienced at temperature higher than 80 °C was about 22.5, 17.6, and 13.5 min, respectively.

Due to the relatively short time experienced to reach the temperature potentially activating the endogenous pectinmethylesterases as well as the relatively long time experienced under temperatures inactivating the enzymes, the extent of pectin de-methylesterification during boiling treatment was supposedly limited versus chemical depolymerization via β-elimination reactions of pectin side chains, thus enhancing cell wall loosening (or rupture) with respect to cell separation when compared against steaming and *sous-vide* treatments. Our hypotheses were in agreement with literature. Constenla and Lozano [44] showed that relatively high temperatures (>80 °C) accelerate the first-order β-eliminative depolymerization of pectin, while pectin de-methylesterification mediated by the pectinmethylesterases follow a second-order kinetics in the range of temperature 50–80 °C with the constant rate increasing exponentially with temperature.

### 3.3. Profiling Extractability of Sterol, Tocopherol, and Water

**Figure 7:**
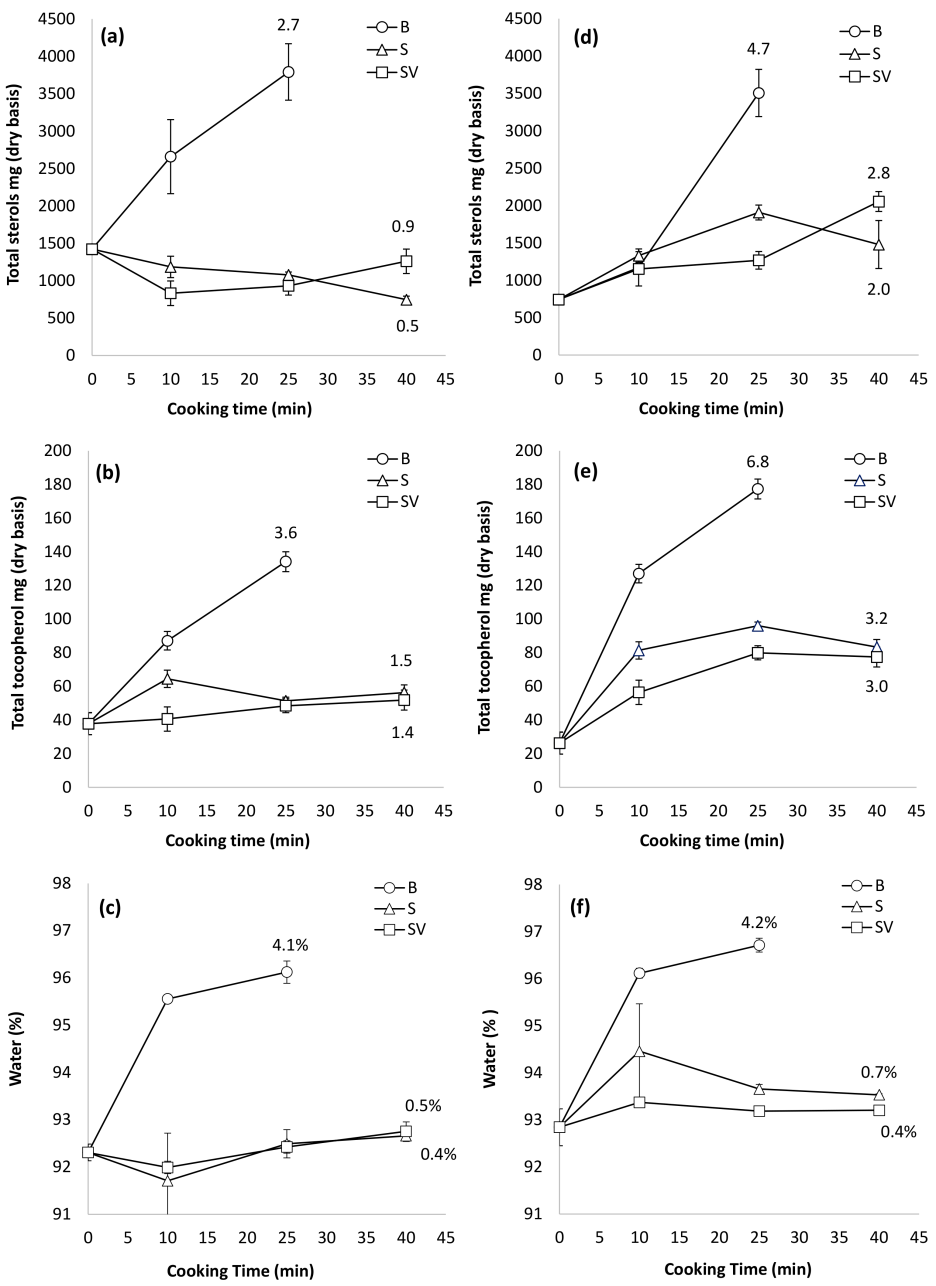
Total sterols (**a**,**d**) and total tocopherol (**b**,**e**) (expressed on dry basis) and percent of water (**c**,**f**) extracted from the *Depurple* (left) and *Cheddar* (right) cauliflower after 10, 25, and 40 min of heating under boiling (B), steaming (S), and *sous-vide* (SV). Error bars represent 95% confidence intervals. Numbers indicated as labels at the end of heating represent the ratio between the concentration of sterols, tocopherols, and water extracted from heated samples with respect to the fresh ones.

Figure 7 compares the total amount of the sterols and tocopherols as well as of the water extracted before and after cooking (error bars represent the standard deviation).

Data related to the sterols and tocopherols were expressed on dry basis, while those referring to the water were expressed as percentage. The water extracted after *sous-vide* treatments was associated with the release of the intercellular fraction of water bound in the middle lamella microdomain due to partial disassembly of the pectin-calcium hydrogel. Otherwise, the increasing in the water extracted after boiling and steaming treatments was associated with the water uptake from the heating medium (hot water and condensed steam, respectively) that was absorbed through hydrogen bonds by the cellulose, hemicellulose. and pectin assemblies in the cell wall microdomain as a function of the extent of degradation of their non-covalent interaction. As it can be argued from Figure 7, the untreated samples belonging to the two varieties of cauliflower were characterized by considerable differences in terms of sterols and tocopherols, with less remarkable but statistically significant differences in terms of the intracellular water. In particular, the concentration of the extractable sterols and tocopherols as expressed on dry basis were 1422 ± 28.3 and 37.8 ± 13.3 mg/kg as well as 745.2 ± 12.5 and 28.5 ± 4.1 mg/kg in *Depurple* and *Cheddar* cauliflower, respectively; while the extracted water was 92.3% ± 0.1%, and 92.8% ± 0.1% in *Depurple* and *Cheddar*, respectively. The highest percentage of water was in agreement with our hypothesis of a higher degree of methylesterification in fresh *Cheddar* with respect to *Depurple* cauliflower: methyl groups are able to hold water molecules via hydrogen bonds. Comparing our results with literature, we found lower levels of sterols in raw cauliflower. In white cauliflower, sterols accounted for 276 mg/kg (wet basis) or 3186 mg/kg (dry basis) [47], 274–411 mg/kg *w*/*w* or 4092–5274 mg/kg (dry basis) [23] and 40 mg/100 g of edible portion [48]. The lower values under our experimental conditions could be due to different extraction method applied: we performed only alkaline saponification of the starting sample, while the authors also performed the acid hydrolysis that is able to release the steryl glycoside sterols. Among vegetables, the highest levels of sterols, e.g., >300 mg/kg (wet basis), were found in broccoli, brussels sprouts, cauliflower, and dill [23]. These higher values were supposed to be due to the higher content of meristematic tissues rich in cell membrane, especially those constituting cauliflower florets [23].

Concerning boiling as the heating technology, the total amount of the extracted sterols, tocopherols as well as of the extracted water increased considerably with the time of heating. After 10 min of heating, the total amount of extracted sterols was on average 1.87 and 1.57 greater than the initial level in the *Depurple* and *Cheddar* cultivar, respectively, while the total amount of tocopherols was on average about 2.3 and 4.62 times greater than the initial level, respectively. After 25 min of heating, the sterols extracted from the two cultivars were about 2.40 and 5.13 times greater than the initial level, while the tocopherols were about 3.55 and 6.43 times, respectively. However, boiling allowed cauliflower tissues to absorb considerable amount of water from heating medium. The water extracted from 10 min boiled samples resulted on average 3.2% and 3.3% greater than the initial level in *Depurple* and *Cheddar* cauliflower, respectively. After 40 min of heating, the extracted water was on average 4.1% and 4.2% greater than the initial level in *Depurple* and *Cheddar* cauliflower, respectively. Such an increased water holding capacity was supposed to be the result of pectin degradation induced by the heat as well as of the changes in the interaction among cellulose, hemicellulose and pectin assemblies. As reviewed by Levesque-Tremblay et al. [41], pectin softening is permissive of hydration. Changes in water holding capacity assume a key meaning also under a nutritional perspective. Referred as wet basis, the level of sterols extracted after 25 min of boiling was on average 1.24 and 2.44 times greater than the initial one for *Depurple* and *Cheddar* cauliflower, respectively. Otherwise, referring on wet basis the tocopherol concentration after 25 min of boiling, the actual increment was on average 1.21 and 3.05 times greater than the initial level for *Depurple* and *Cheddar* cauliflower, respectively.

Literature on sterol refers only to white cauliflower and boiling cooking. Kaloustian et al. [47] claimed that 30 min of boiling increased the level of the free sterols in eight plant products, including cauliflower, if dry matter was considered. They found a double increment of extractable sterols (from 3186 to 6250 mg/kg expressed in dry basis) in boiling fresh cauliflower. The authors also concluded that cooked vegetables might give better protection against cardiovascular diseases due to higher sterol levels. Normén et al. [48] found in boiled white cauliflower a decreasing level of extractable sterols with no significant differences with respect to other investigated vegetables (in this work the time of boiling was not specified). To the best of our knowledge, no literature is available on sterol extractability with respect to cooking technologies different from boiling (e.g., steaming and sous-vide).

Concerning steaming as heating technology, the total amount of the extracted sterols decreased at each time of heating in the *Depurple* samples (Figure 7a, differences were significant with *p* < 0.05), reaching after 40 min on average about 0.50 times the initial level. Under our experimental conditions, the total amount of sterols increased with the time of heating in *Cheddar* cauliflower, reaching after 40 min on average 2.32 times greater than the initial level (differences were significant with *p* < 0.05). Conversely, the total amount of tocopherols extracted after 10 min of heating from was on average 1.70 and 2.97 times the initial level (Figure 7b,e) in the *Depurple* and *Cheddar* samples, respectively. After 25 min of heating, the total amount of extracted tocopherols was on average 1.36 and 3.49 times the initial level in the *Depurple* and *Cheddar* samples, respectively. After 40 min of steaming, the total amount of extracted tocopherols was 1.50 and 3.20 times the initial level in the *Depurple* and *Cheddar* samples, respectively. Concerning the total amount of the water extracted after 10 min of steaming resulted on average of about 0.2% lower and 1.7% greater than the initial level in the *Depurple* and *Cheddar* samples, respectively. The pronounced absorption of water was in agreement with our hypothesis of a higher degree of pectin methylesterification in *Cheddar* cauliflower. However, the high variability observed in the percentage of the water extracted at 10 min of steaming (Figure 7c,f) suggested that this cooking technology resulted in non-uniform heating effects in the early steps of cooking in both the cauliflower varieties. An additional time of steaming, i.e., from 25 to 40 min, permitted a less considerable increase of extracted water, i.e., on average of about 0.5% in the *Depurple* and 0.7% in *Cheddar* samples, resulting in reduced variability. As reviewed by Xiao et al. [49], although steaming causes weight loss less pronounced with respect to boiling, reducing leaching of nutrients in the condensed medium, and the formation of a dried layer on product surface due to evaporation of water, authors concluded that steaming results in non-uniform heating effects and needs longer treatment time than hot water, due to the lower heat transfer in steam than in hot water.

Concerning *sous-vide* as heating technology, the total amount of the extracted sterols from *Depurple* samples decreased considerably within 10 min of heating, then it moderately increased after 25 and 40 min of heating (*p* < 0.05). However, the sterols extracted after 40 min of heating were 0.9 times the initial level (untreated samples). Otherwise, the sterols extracted from *Cheddar* samples increased considerably at each time of heating: After 40 min it resulted on average 2.8 times greater than the initial level. The total amount of the extracted tocopherols after 40 min of heating increased moderately in *Depurple* samples and considerably in the *Depurple* samples: the final level was 1.4 and 3.0 times greater than the initial one (*p* < 0.05), respectively. The total amount of water extracted from *Depurple* samples heated under *sous-vide* undergo changes close to that observed in the same variety as heated under steaming. However, the amount of water extracted from *Cheddar* samples was relatively lower after each time of heating, with a significant increase after 10 min (*p* < 0.05).

Several studies suggested that considerable differences in compounds extractability among vegetables can be associated with the morphological structure and tissue thickness [4].

In this study, the negative correlation of sterols extractability with time of steaming and *sous-vide* in *Depurple* and the positive correlation in *Cheddar* cauliflower were explained supposing variety-dependent differences in pectin methylesterification and microstructure at a cellular scale. As reviewed by Liu et al. [36] the effects of processing on pectin structure are highly dependent on the processing conditions: pectin with low degree of methylesterification caused lower bioaccessibility of both lipophilic and hydrophilic bioactive compounds as compared to pectin with high degree of methylesterification, which may be attributed to higher pectin binding capacities. Localized stiffness in cell wall microdomains and in cell membrane were also proposed as two additional variety-related factors affecting functional properties of the cell wall, in the sense of its effectiveness in decompartmentalize sterols as affected by heat: local heterogeneity affected pectin degradation unevenly throughout primary cell wall microdomain thus explaining the apparent synchrony in terms of compliances (as described by **U_r_**, **J_R_**, and **J_N_** in Figure 5) and the apparent asynchrony in terms of extractability of sterols and tocopherols under steaming and *sous-vide* (as reported in Figure 7a,b,d,e). A non-homogeneous distribution of linear pectin of various methylesterification degrees and patterns is documented in literature as a key factor contributing to spatial differences in the elasticity, hydration, porosity, and adhesion properties, either at a cell wall scale and tissue scale. As reviewed by Levesque-Tremblay et al. [41], as high as 80% of homogalacturonan (linear) pectin in growing cells is methyl esterified before its secretion from Golgi apparatus to cell wall where it redistributes and concentrates locally after de-methylesterification mediated by endogenous pectinmethylesterases. De-methylesterified homogalacturonan may encounter two general fates: (1) the formation of a stable structure with other homogalacturonan molecules (causing local strengthening of the cell wall), or (2) degradation by polygalacturonases (causing local loosening of the cell wall). As reviewed by Turner and Kumar [25], sterols may play an indirect role in cellulose biosynthesis enabling the processes of acylation of targeted membrane proteins and of membrane partitioning into sterol-rich microdomains in the region of cellulose synthesis. As consequence, local heterogeneity in the cell wall and cell membrane microstructure may be expected as plant variety-specific properties, thus providing a possible explanation because sterol extractability decreased in *Depurple* and increased in *Cheddar* when they were treated under lower heat transfer efficiency conditions, i.e., steaming and *sous-vide*.

The extractability profiling was analyzed also with respect to the main differences observed in terms of the individual active compounds. The level of individual sterols and tocopherols are reported in Table 2 (mg/kg of dry basis) and Table 3 (mg/kg of wet basis) together with their 95% confidence limits.

As it can be argued from data, in all samples (untreated and cooked) β-sitosterol was the major sterol followed by campesterol and stigmasterol. *Depurple* cauliflower was 2-fold richer in campesterol, stigmasterol and β-sitosterol than *Cheddar*. Our hypothesis of a lower degree of the methylesterification and higher compliance in fresh *Depurple* cauliflower was corroborated by the corresponding higher concentration of sterols, mainly β-sitosterol and campesterol (Table 2 and Table 3), in the cell membrane. Da Silveira et al. [50] reported that high sterol content (with a decreasing order between β-sitosterol, campesterol and stigmasterol) is related to an increment of the cell membrane thickness and of turgor. Greve et al. [37] showed that a higher level of turgor in fresh carrots causes more rapid loss in firmness during the early boiling (1 min).

Expressing data on fresh vegetables, the major positive effect on phytosterols levels in violet cauliflower was recorded after boiling (10 and 25 min with no statistical difference between minutes) and the major negative effect after steaming at 40 min and sous-vide cooking at 10 min.

### 3.4. Interplay between Phytochemical Extractability, Cell Separation and Cell Wall Rupture

The profiles of the total sterols and tocopherols extracted after cooking were analyzed as a function of the impact of heat on tissue microstructure as described by the rheological parameters determined in the range of both the large and small scale of deformation.

Concerning the range of large-scale deformation (macroscopic scale), the loss of the stiffness in *Cheddar* cauliflower (Figure 2) after 25 min of *sous-vide*, steaming and boiling, were coherent with the increasing in total concentration of sterols and tocopherols extracted after cooking. The amount of the individual sterols and tocopherols are reported in Table 2 and Table 3. Sterols were 1269.3 ± 203.2, 1908.5 ± 172.8, and 3822.0 ± 57.8 mg/kg, respectively, while tocopherols were 83.3 ± 10.2, 99.7 ± 16.5, and 183.4 ± 24.1 mg/kg, respectively. Results support the idea that the ease to extract the two classes of bioactive macromolecules is strictly related to thermal softening on a macroscopic scale. However, for extractability to increase, hierarchical disassembling of tissue microstructure is required on a microscopic scale, through different potential mechanisms which include cell membrane disruption and releasing of molecules entrapped in the phospholipid by-layer, pectin degradation extending from middle lamella (causing cell separation) to cell wall loosening (causing increasing of porosity or loss in the cell wall thickness). Prevalence of cell wall loosening to cell separation mechanisms must be considered as the main cause of the increase of macromolecules extractability and of their bioaccessibility during consumption.

With the aim to investigate the impact of cooking in terms of the tissue decompartmentalization and its relation on the extractability of sterols and tocopherols, two microstructure-related parameters were defined using the rheological parameters determined in the range of small scale of deformation, namely the “Elastic Recovery Ability, **ERA**” and the “Elastic-to-Viscoplastic Ratio, **EPR**”.

The **ERA** parameter was calculated as in the following:

(3) $$\mathrm{ERA}=100\;\cdot \frac{J_{\max}{\;-\;J}_{\mathrm{eq}}}{J_{\max}}$$

**ERA** was used as a descriptor of the recoverable elasticity at a cell wall scale, thus positively related to the ability to bear turgor pressure, and negatively to the phytochemicals extractability. **ERA** may vary from 100 (complete recovery of microstructure elasticity) to 0 (complete loss of microstructure elasticity). Decreasing in the **ERA** parameter as induced by heat were treated as indirect measure of the residual cell wall integrity due to the cumulative damage in terms of cell membrane disassembling (loss of turgor) and irreversible pectin degradation in both the cell wall and middle lamella microdomains.

The **EPR** parameter was calculated as in the following:

(4) $$\mathrm{EPR}=\;\frac{J_{0}+\sum_{1}^{3}J_{i}}{J_{N}}\;\;$$

**EPR** was used as a descriptor of the prevalence of the disassembling mechanisms causing cell wall loosening to those causing cells separation without cell rupture, thus positively related to the easy to extract sterols and tocopherols.

Values of **ERA** and **EPR** are reported in Table 1. As can be argued from data, the two varieties showed the same initial ability to prevent cell loosening and cell separation mechanisms as indicated by the level of **EPR** in the untreated samples belonging to the two varieties (difference was not significant, *p* > 0.05). However, they were characterized by different type of interaction among the cell wall polymers, and therefore different ability to counterbalance turgor pressure and to resist pectin degradation during boiling, steaming, and *sous-vide*. The untreated *Depurple* cauliflower showed the highest level of **ERA**. Moreover, **ERA** decreased continuously over cooking with an increasing order under *sous-vide*, steaming and boiling conditions. The **ERA** decreasing rate was in *Cheddar* cauliflower greater than in the *Depurple* one. Such results suggested that fresh *Depurple* cauliflower was characterized by strengthen cell wall with an ability to bear turgor pressure and resist pectin degradation under heating higher than in the *Cheddar* cauliflower. Changes in polymer interaction during heating affected differently the two cultivars to resist cell loosening and cell separation. The level of **EPR** registered under boiling and steaming conditions reached a minimum value at 10 min of heating after which it increased in *Cheddar* cauliflower with a rate greater than in the *Depurple* one, thus highlighting a variety-specific ability to shift mechanisms causing cell separation to cell wall loosening.

**Figure 8:**
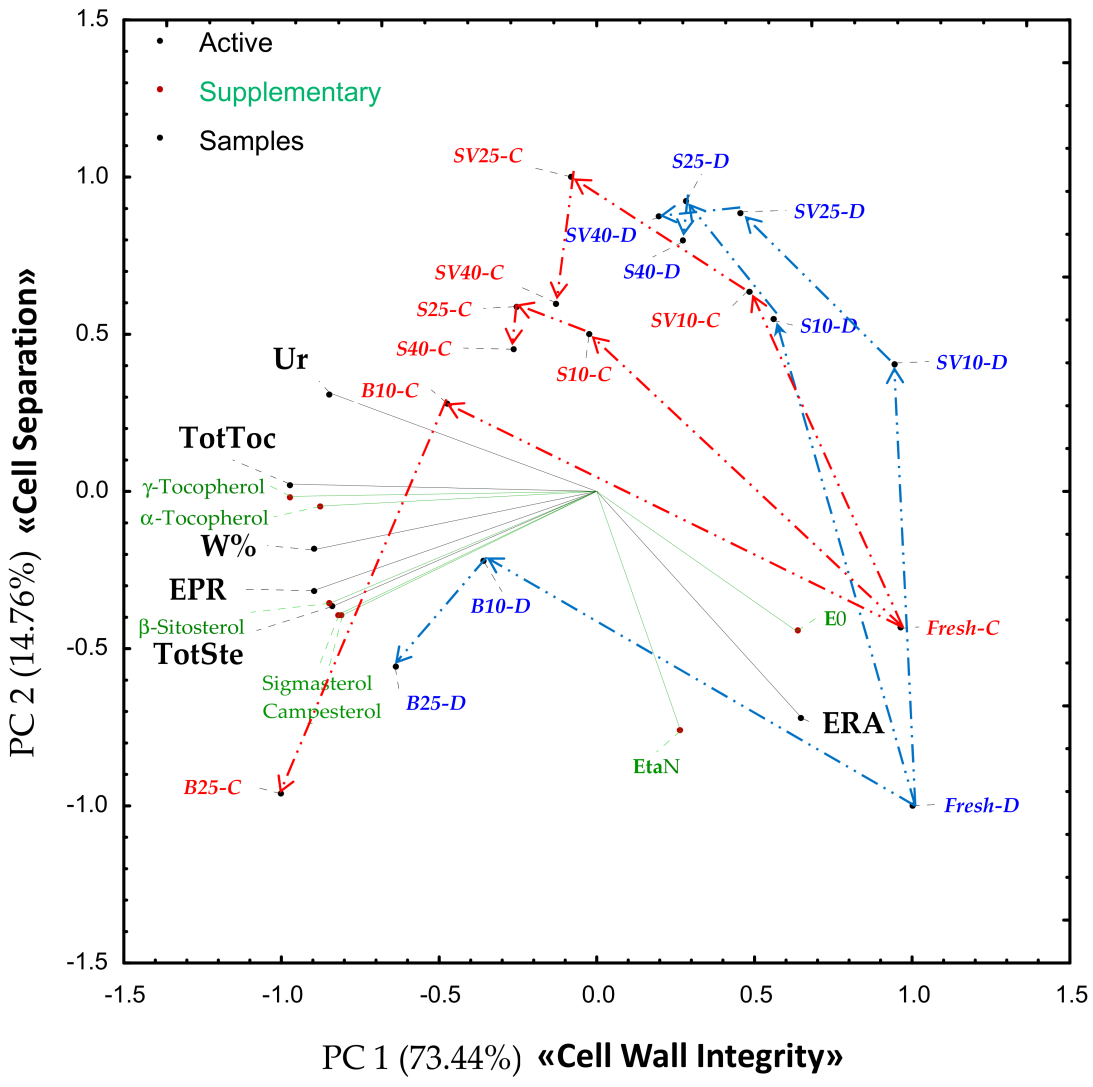
Results from principal component analysis (PCA) performed using the concentration (on dry basis) of total sterols and tocopherols and certain rheological properties as input variables to obtain insight on the impact of the heat in *Depurple* (blue) and *Cheddar* (red) cauliflower during steaming (S), *sous-vide* (SV), and boiling (B), on the phytochemical extractability. S10, S25, and S40 indicate 10, 25, and 40 min of exposure to steaming in the saturated oven; SV10, SV25, and SV40 indicate 10, 25, and 40 min of exposure to sous-vide in the saturated oven; B10 and B25 indicate 10 and 25 min of exposure to boiling in the hot water; D and C suffixes in the labels represent the *Depurple* and *Cheddar* variety, respectively; TotSte, TotToc are the total amount of sterols (campesterol, β-sitosterol, and stigmasterol) and tocopherols (α-tocopherol and γ-tocopherol), respectively; **ERA** is the elastic recovery ability (it is used as an indirect measure of the loss of cell membrane and cell wall integrity) calculated as $\mathrm{ERA}=100\;\cdot \left[\left(J_{\max}{\;-\;J}_{\mathrm{eq}}\right){/J}_{\max}\right]$, where J_max_ and J_eq_ are the maximum creep compliance and the equilibrium compliance at the end of the recovery step, respectively; **EPR** is the elastic-to-plastic ratio (it is used as an indirect measure of the loss of cell membrane and cell wall integrity) calculated as ${\mathrm{EPR}=[J}_{0}+\overset{3}{\underset{1}{\sum }}J_{i}{]/J}_{N}$, where **J_0_** and **J_i_** are the instantaneous and the three retarded components of creep compliance in the tailored Burger’s model, respectively.

To obtain insight on the interplay relating cooking technology, heating time, tissue softening, cell wall loosening and phytochemical extractability, the parameters **ERA**, **EPR**, and **U_r_** together with the total amount of sterols (**TotSte**) and of tocopherols (**TotToc**) as well as the percentage of water extracted (**W%**) were treated as active variables in a principal component analysis (PCA). Campesterol, stigmasterol, β-sitosterol as well as γ-tocopherol and α-tocopherol together with the **EtaN** and **E_0_** parameters were treated as supplementary variables aiming to full characterize the samples under cooking. Results from PCA are reported in the Figure 8, in which the samples belonging to *Depurple* variety are highlighted in blue and those belonging to *Cheddar* in red.

The score loadings corresponding to the samples treated under the same cooking technology were also connected with consecutive arrows to trace the pattern of microstructural changes during heating (from 0 to 40 min). As can be argued from figure, the pattern of changes was similar under the same cooking technology, but satisfactorily discriminated the samples belonging to *Depurple* variety from those belonging to the *Cheddar* one. The first three principal components explained more than 93% of the experimental variance (73.44%, 14.76%, and 11.8%, respectively). The **ERA** parameter was positively correlated to **E_0_** and opposite to **EPR** and **U_r_**. **EPR** was also opposite to **U_r_** and **EtaN**. Such relationships corroborated the idea that the covalent bonds (as indicated by **E_0_**) are positively linked to both the turgor pressure and cell wall integrity (as indicated by **ERA**), and that cell separation (as indicated by low levels of **EPR**) is the main cause of softening (as expressed in terms of **U_r_**) resulting in a decrease of non-covalent bonds (as indicated by low levels in **EtaN**). According to the magnitude and signs of the factor coordinates of the two microstructural-related parameters **ERA** and **EPR**, the first two principal components (PC 1 and PC 2) were meaningfully labeled as to “Cell Wall Integrity” and “Cell Separation”, respectively. Magnitude and signs of the factor coordinates also indicated that **TotToc**, **TotSte**, and **W%** were positively correlated to **EPR** and **U_r_** as well as that they were negatively correlated to **ERA**. Such results provided compelling evidence that the extractability of sterols and tocopherols was in agreement with the loss of cell wall integrity (as described by **ERA**) and that it was in *Cheddar* cauliflower higher than in *Depurple*. The extractability of the phytochemicals increases with the time of heating to such an extent depending on the ability of the cooking technology to enhance cell wall loosening to a greater extent with respect to the viscous flow in the middle lamella through cell separation, thus achieving low levels of **ERA** and high levels of **EPR**.

The time-dependent pattern of changes in terms of loss of cell wall integrity rather than cell separation can be advantageously analyzed by the range of variation of the two principal components with respect to the time of cooking.

PC 1 assumed positive sign throughout the entire period of heating under steaming and *sous-vide*, while it decreased from positive to negative values under boiling.

Additively, the range of variation of PC 1 was always lower in *Depurple* cauliflower than that characterizing *Cheddar*, most likely due to the nonhomogeneous distribution of pectin degradation at a local scale in under low efficiency in both heat transfer conditions. The range of variation of PC1 followed the decreasing order under boiling, steaming and sous-vide. The opposite can be observed for the PC 2. This reflects the idea that boiling was the most effective cooking method to enhance the mechanisms causing tissue decompartmentalization through cell wall loosening (a, b) with respect to those causing cell separation (c, d) having no impact on the phytochemical extractability. *Sous-vide* showed the lowest impact on cell membrane and cell wall integrity, but the highest in terms of cell separation. Steaming showed an intermediate behavior.

Table 4 lists the multiple regression models able to predict the concentration of sterols, tocopherols (on dry basis) that can be extracted from cauliflower after cooking by using **ERA** and **EPR** as input predictors. The regression coefficients are reported together with their standard errors.

### 3.5. Decompartmentalization Kinetics

Based on the results from PCA, the **ERA** parameter was considered the most significant microstructure-related parameter positively related at cell scale to the extractability of the sterols and tocopherols and negatively related to the softening parameter (**U_r_**). Thus, a kinetic model was proposed treating the **ERA** parameter as a dependent variable to describe the kinetics of decompartmentalization positively related to the increase of phytochemical extractability during heating. With this aim, changes in the **ERA** parameter were numerically analyzed as a function of time of heating to determine the *characteristic fixed-order* (**n**) of the softening kinetics and its *characteristic constant rate* (**k**) under each investigated cooking condition. The following decay function was fitted to the experimental data:

(5) $$\mathrm{ERA}\left(t\right){=\mathrm{ERA}}_{0}+\left[{\mathrm{ERA}}_{0}\;-{\mathrm{ERA}}_{\infty }\right]\cdot \exp\left(-\;k\cdot t^{n}\right)$$

where ${\mathrm{ERA}}_{0}$, $\mathrm{ERA}\left(t\right)$, and ${\mathrm{ERA}}_{\infty }$ are the decaying levels of **ERA** assumed before heating (referred to the untreated sample), after a given time (t) of heating, and after a long time (infinity) of heating, respectively; **n** is adimensional and **k** is expressed in min**^−1^**. Application of Equation (5) necessitated the prior knowledge of the equilibrium values of the rheological parameters in each of the investigated cooking conditions. Since it was not possible to determine the value of ${\mathrm{ERA}}_{\infty }$ experimentally in all creep experiments due to the fragile nature of the cauliflower samples, especially after prolonged heating, the values of the parameters **k**, **n** and ${\mathrm{ERA}}_{\infty }$ were estimated simultaneously by nonlinear regression analysis.

**Figure 9:**
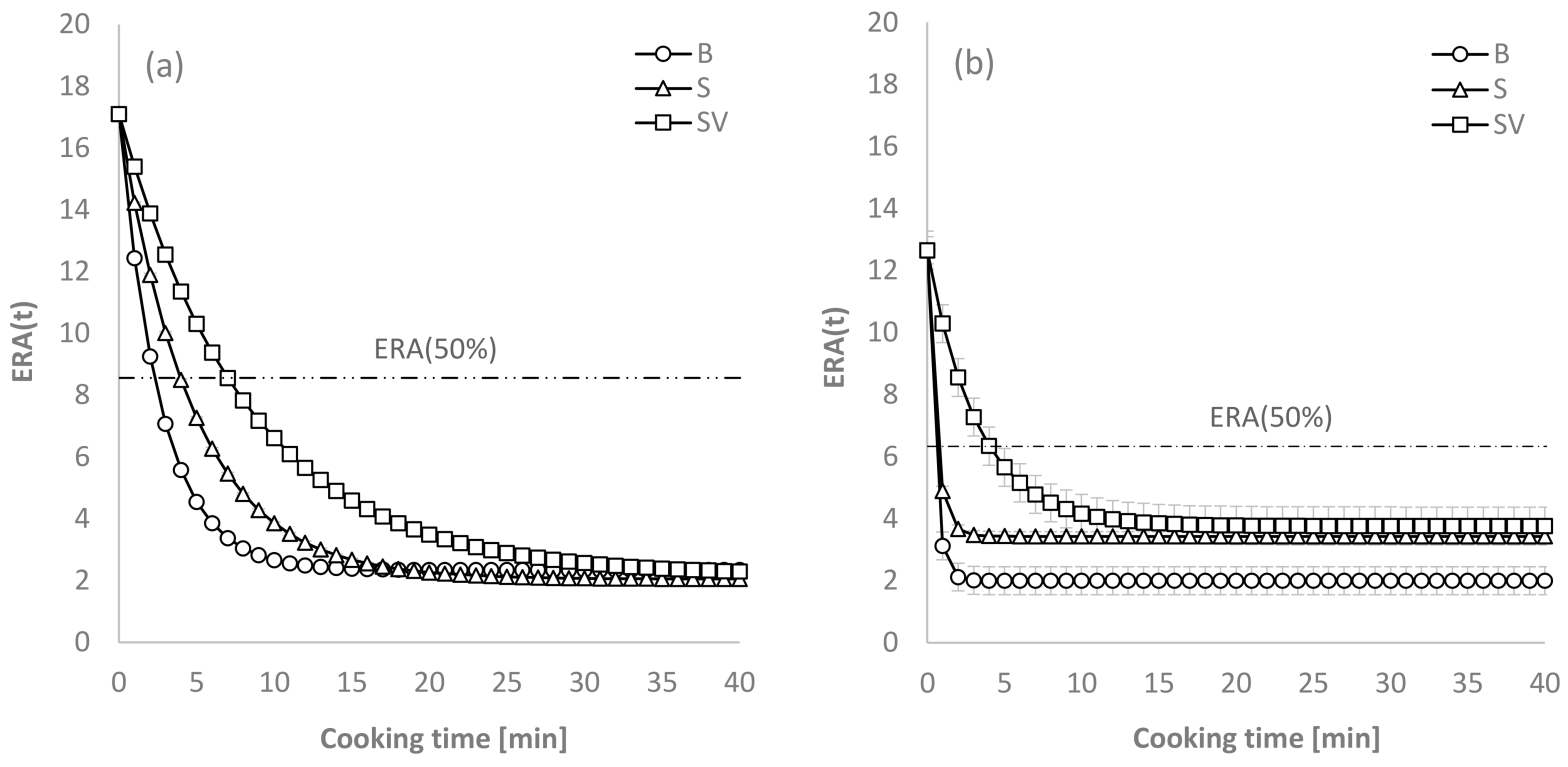
Tissue decompartmentalization kinetics of *Depurple* (**a**) and *Cheddar* (**b**) cauliflower under boiling (B), steaming (S), and *sous-vide* (SV) as described by the ERA parameter (intended as an indirect measure of the loss of cell wall integrity and of the easy of extractability of sterols and tocopherols).

Results from the regression analysis of the kinetic model on the experimental data are reported in Figure 9.

The three kinetic parameters were estimated by performing a non-linear fitting procedure of Equation (5) on the experimental data (**N**). The lack-of-fit was evaluated by calculating the square relative mean error (**E%**, always less than 2%):

(6) $$\tilde{E}\%=\frac{100}{N-3}\cdot \sqrt{\frac{{\left[\mathrm{ERA}\left(t\right)-\mathrm{ERA}{\left(t\right)}_{\exp}\right]}^{2}}{\mathrm{ERA}\left(t\right)}}$$

As it can be inferred from the data, tissue decompartmentalization followed an apparent first-order decay kinetics (**n** = 1), irrespective of cooking technology and cauliflower variety. For comparison purposes, the constant rate k, ${\mathrm{ERA}}_{\infty }$ and the characteristic decay time, in the figure referred as to “t_50_”, indicating the time of heating required to reach 50% of the initial level of **ERA** (fresh samples), were reported in Figure 9 together with the determination coefficient R^2^ of the linear regression of predicted vs. observed with a statistical significance of *p* < 0.05.

Provided that a validation study will be required to establish an acceptable level of softening (residual texture) after cooking for a given cauliflower variety, the proposed kinetic model can be of usefulness under the optimization perspective, to simulate the time of heating under a specified cooking technology aiming to maximize the extractability of sterols and tocopherols by keeping an acceptable level of softening.

## 4. Conclusions

Is this study, the effects induced by heat on two varieties (*Depurple* and *Cheddar)* of cauliflower were investigated under boiling, steaming, and *sous-vide*.

An elastoplastic model based on hypothesis structure-function relationships among the cellulose, hemicellulose, and pectin assemblies in the cell wall and middle lamella microdomains was derived by tailoring a Burger’s function to describe creep behavior in the range of small-scale compliance through cell wall loosening to cell separation mechanisms, directly related to the ease of extractability of sterols and tocopherols

Results showed that campesterol, stigmasterol, β-sitosterol, α-tocopherol and γ-tocopherols can become “potentially bioaccessible” with the decreasing order after boiling, steaming, and *sous-vide* treatments. Differences in their extractability of sterols and tocopherols were associated with the selective ability of the actual time-temperature conditions in promoting cell rupture with respect to cell separation, *cauliflower variety* as well as *chemical nature of the specific compound*. Local heterogeneity in the cell wall and cell membrane microstructure, expected as a plant variety-dependent functional property, was proposed as a possible explanation because the increase of the sterol extractability under lower heat-transfer efficiency, i.e., steaming and *sous-vide*, was lower in *Depurple* than in *Cheddar* during early heating as well as because the extractability of sterols and tocopherols was greater in *Cheddar* during late heating.

A kinetic model was also proposed as a practical tool to describe the tissue decompartmentalization kinetics coupled with the extractability of sterols and tocopherols, enabling maximization of their extractability by keeping an acceptable level of softening. 

## Figures and Tables

**Table 1:** Impact of the boiling (B), steaming (S) and sous-vide (SV) on the rheological properties on a microscopic scale as described by the parameters obtained from the tailored Burger’s model for *Depurple* (top) and *Cheddar* (bottom) cauliflower.

	Time	J_0_	J_N_	J_1_	J_2_	J_3_	J_max_	J_eq_	EtaN	J_R_	U_r_	ERA	EPR
	min	kpa^−1^	kpa^−1^	kpa^−1^	kpa^−1^	kpa^−1^	kpa^−1^	kpa^−1^	kPa*s	kpa^−1^	(s/kPa)10^2^	%	adim
*Depurple*													
Fresh	0	6.3 × 10^−3^	3.0 × 10^−6^	2.3 × 10^−2^	5.3 × 10^−3^	3.8 × 10^−3^	3.0 × 10^−2^	2.5 × 10^−2^	3.4 × 10^5^	2.7 × 10^−2^	2.7 × 10^−1^	17.21	21.41
S	10	1.0 × 10^−2^	7.9 × 10^−6^	2.7 × 10^−2^	1.0 × 10^−2^	1.0 × 10^−2^	5.0 × 10^−2^	4.8 × 10^−2^	1.1 × 10^5^	4.0 × 10^−2^	2.9 × 10^−1^	3.88	12.18
S	25	1.2 × 10^−2^	6.7 × 10^−6^	3.5 × 10^−2^	1.2 × 10^−2^	7.8 × 10^−3^	7.3 × 10^−2^	7.1 × 10^−2^	1.5 × 10^5^	4.7 × 10^−2^	5.8 × 10^−1^	2.26	16.45
S	40	1.4 × 10^−2^	6.0 × 10^−6^	2.8 × 10^−2^	1.1 × 10^−2^	7.8 × 10^−3^	6.5 × 10^−2^	6.2 × 10^−2^	1.7 × 10^5^	4.4 × 10^−2^	6.2 × 10^−1^	4.51	16.91
SV	10	7.9 × 10^−3^	8.2 × 10^−6^	3.8 × 10^−2^	1.3 × 10^−3^	9.5 × 10^−3^	4.3 × 10^−2^	4.1 × 10^−2^	1.2 × 10^5^	2.8 × 10^−1^	2.8 × 10^−1^	6.68	11.46
SV	25	1.1 × 10^−2^	7.3 × 10^−6^	3.5 × 10^−2^	1.8 × 10^−3^	9.6 × 10^−3^	7.9 × 10^−2^	7.7 × 10^−2^	1.4 × 10^5^	4.8 × 10^−1^	4.8 × 10^−1^	2.68	13.04
SV	40	1.3 × 10^−2^	6.9 × 10^−6^	3.8 × 10^−2^	1.2 × 10^−3^	1.0 × 10^−2^	8.3 × 10^−2^	8.1 × 10^−2^	1.5 × 10^5^	6.1 × 10^−1^	6.1 × 10^−1^	2.45	12.88
B	10	1.2 × 10^−2^	5.7 × 10^−6^	5.0 × 10^−2^	1.1 × 10^−2^	8.1 × 10^−3^	8.8 × 10^−2^	8.6 × 10^−2^	1.8 × 10^5^	5.5 × 10^−2^	6.8 × 10^−1^	2.74	23.78
B	25	1.5 × 10^−2^	4.6 × 10^−6^	5.1 × 10^−2^	1.6 × 10^−2^	6.5 × 10^−3^	6.5 × 10^−2^	6.3 × 10^−2^	2.2 × 10^5^	6.1 × 10^−2^	7.7 × 10^−1^	2.34	31.71
*Cheddar*													
Fresh	0	4.1 × 10^−3^	2.2 × 10^−6^	1.6 × 10^−2^	5.7 × 10^−3^	3.1 × 10^−3^	4.9 × 10^−2^	4.2 × 10^−2^	4.6 × 10^5^	2.0 × 10^−2^	2.9 × 10^−1^	12.54	22.53
S	10	8.7 × 10^−3^	8.8 × 10^−6^	3.4 × 10^−2^	1.2 × 10^−2^	9.9 × 10^−3^	8.7 × 10^−2^	8.4 × 10^−2^	1.1 × 10^5^	4.4 × 10^−2^	4.8 × 10^−1^	3.18	12.23
S	25	1.3 × 10^−2^	6.3 × 10^−6^	4.9 × 10^−2^	9.4 × 10^−3^	7.9 × 10^−3^	9.7 × 10^−2^	9.4 × 10^−2^	1.6 × 10^5^	5.5 × 10^−2^	9.6 × 10^−1^	3.32	21.09
S	40	1.7 × 10^−2^	5.3 × 10^−6^	5.2 × 10^−2^	8.9 × 10^−3^	6.6 × 10^−3^	8.7 × 10^−2^	8.4 × 10^−2^	1.9 × 10^5^	6.0 × 10^−2^	1.0 × 10^0^	3.82	26.91
SV	10	5.7 × 10^−3^	9.9 × 10^−6^	3.2 × 10^−2^	1.0 × 10^−2^	1.0 × 10^−2^	7.3 × 10^−2^	7.0 × 10^−2^	1.0 × 10^5^	3.9 × 10^−2^	3.9 × 10^−1^	4.20	9.85
SV	25	9.4 × 10^−3^	7.4 × 10^−6^	4.6 × 10^−2^	1.0 × 10^−2^	8.6 × 10^−3^	8.4 × 10^−2^	8.2 × 10^−2^	1.4 × 10^5^	5.0 × 10^−2^	8.0 × 10^−1^	2.33	16.79
SV	40	1.5 × 10^−2^	6.6 × 10^−6^	2.6 × 10^−2^	2.0 × 10^−2^	1.1 × 10^−2^	7.9 × 10^−2^	7.5 × 10^−2^	1.5 × 10^5^	5.2 × 10^−2^	9.5 × 10^−1^	5.17	18.28
B	10	9.2 × 10^−3^	4.4 × 10^−6^	6.2 × 10^−2^	9.8 × 10^−3^	6.4 × 10^−3^	8.5 × 10^−2^	8.3 × 10^−2^	2.3 × 10^5^	5.9 × 10^−2^	8.5 × 10^−1^	1.75	33.46
B	25	1.4 × 10^−2^	3.4 × 10^−6^	8.3 × 10^−2^	1.0 × 10^−3^	4.4 × 10^−3^	1.0 × 10^−1^	9.9 × 10^−2^	2.9 × 10^5^	5.7 × 10^−2^	9.9 × 10^−1^	2.25	49.95

S, SV, and B indicate steaming, sous-vide and boiling, respectively; **J_0_** (kPa^−1^) is the instantaneous reversible compliance associated with the elastic stretching of the covalent bonds having the highest degree of freedom in the linear polymer fractions present in the cellulose microfibril network; **J_1_**, **J_2_**, and **J_3_** (kPa^−1^) are the retarded reversible compliances of the three main viscoelastic elements in the cellulose-hemicellulose-pectin network; **J_N_** (kPa^−1^) is the steady-state irreversible compliance (with **EtaN** (kPa∙s) = 1/**J_N_**); **U_r_** (s/KPa) is the integrated compliance calculated as numerical integral from zero to the time at load removing of the creep compliance curve (it represents the energy releasing rate under creep); **ERA** is the elastic recovery ability at cell scale (it is used as an indirect measure of the loss of cell membrane and cell wall integrity) calculated as $\mathrm{ERA}=100\;\cdot \left[\left(J_{\max}{\;-\;J}_{\mathrm{eq}}\right){/J}_{\max}\right]$, where **J_max_** and **J_eq_** are the maximum creep compliance and the equilibrium compliance at the end of the recovery step, respectively.

**Table 2:** Sterols and tocopherols extracted from *Depurple* cauliflower before (fresh) and after heating under boiling (B), steaming (S), and *sous-vide* (SV). Data are expressed as dry basis (top, mg/kg dw) and wet basis (bottom, mg/kg *w*/*w*).

Sample	Campesterol	Stigmasterol	β-sitosterol	Total sterols	α-tocopherol	γ-tocopherol	Total tocopherols
	mg/kg	mg/kg	mg/kg	mg/kg	mg/kg	mg/kg	mg/kg
*Dry basis*							
Raw	290.9 ± 20.6 ^b^	54.1 ± 6.0 ^a^	1077.1 ± 10.1 ^c^	1422.0 ± 28.3 ^d^	11.8 ± 2.0 ^a^	26.0 ± 9.5 ^a^	37.8 ± 11.3 ^a^
S10	252.5 ± 33.0 ^b^	37.2 ± 4.6 ^a^	1020.3 ± 134.6 ^bc^	1310.0 ± 163.0 ^cd^	17.6 ± 2.1 ^bc^	46.9 ± 10.7 ^ab^	64.5 ± 12.6 ^ab^
S25	209.9 ± 15.8 ^ab^	25.5 ± 6.5 ^a^	841.0 ± 54.4 ^b^	1076.3 ± 68.5 ^c^	15.0 ± 1.0 ^ab^	36.3 ± 6.4 ^a^	51.4 ± 7.3 ^a^
S40	144.4 ± 19.6 ^a^	25.8 ± 3.0 ^a^	576.9 ± 63.6 ^a^	747.2 ± 80.9 ^a^	15.8 ± 2.5 ^ac^	40.6 ± 7.8 ^a^	56.3 ± 10.3 ^a^
SV10	122.5 ± 33.1 ^a^	22.0 ± 24.1 ^a^	551.2 ± 162.8 ^a^	695.6 ± 219.9 ^a^	12.8 ± 1.8 ^ab^	27.8 ± 7.7 ^a^	40.6 ± 8.9 ^a^
SV25	215.8 ± 11.9 ^ab^	14.6 ± 11.8 ^a^	824.2 ± 55.5 ^b^	1054.6 ± 79.2 ^abc^	14.5 ± 0.6 ^ab^	34.1 ± 3.7 ^a^	48.5 ± 3.9 ^a^
SV40	292.0 ± 4.4 ^b^	53.7 ± 12.2 ^a^	1076.9 ± 13.8 ^bc^	1422.6 ± 2.8 ^bd^	15.6 ± 2.1 ^abc^	36.2 ± 5.7 ^a^	51.8 ± 7.8 ^a^
B10	538.8 ± 27.2 ^c^	104.1 ± 2.9 ^b^	2025.6 ± 11.7 ^d^	2668.5 ± 41.8 ^c^	20.8 ± 1.3 ^c^	66.3 ± 8.3 ^b^	87.1 ± 9.5 ^b^
B25	688.2 ± 71.4 ^d^	131.0 ± 23.6 ^b^	2598.1 ± 27.4 ^e^	3417.4 ± 122.4 ^f^	29.7 ± 2.8 ^d^	104.5 ± 7.5 ^c^	134.1 ± 10.2 ^c^
*Wet basis*							
Raw	22.4 ± 2.4 ^bc^	4.2 ± 0.6 ^ac^	82.8 ± 3.1 ^cd^	109.4 ± 6.1 ^bc^	0.9 ± 0.2 ^a^	2.0 ± 0.8 ^a^	2.9 ± 1.0 ^a^
S10	18.1 ± 1.7 ^abc^	2.7 ± 0.4 ^ac^	73.1 ± 7.0 ^bcd^	93.9 ± 8.3 ^abc^	1.5 ± 0.3 ^b^	4.0 ± 1.5 ^ab^	5.4 ± 1.8 ^b^
S25	15.7 ± 0.3 ^ab^	1.9 ± 0.5 ^a^	62.9 ± 0.2 ^abc^	80.6 ± 0.9 ^ab^	1.1 ± 0.1 ^ab^	2.7 ± 0.4 ^ab^	3.8 ± 0.4 ^ab^
S40	10.6 ± 1.7 ^a^	1.9 ± 0.2 ^a^	42.4 ± 5.9 ^a^	54.9 ± 7.5 ^a^	1.2 ± 0.2 ^ab^	3.0 ± 0.6 ^ab^	4.1 ± 0.8 ^ab^
SV10	9.7 ± 2.8 ^a^	1.8 ± 1.9 ^ab^	43.6 ± 13.7 ^ab^	55.1 ± 18.4 ^a^	1.0 ± 0.1 ^ab^	2.2 ± 0.6 ^ab^	3.3 ± 0.8 ^ab^
SV25	16.3 ± 0.2 ^ab^	1.1 ± 0.8 ^a^	62.2 ± 1.6 ^abc^	79.5 ± 2.6 ^ab^	1.1 ± 0.1 ^ab^	2.6 ± 0.2 ^ab^	3.7 ± 0.2 ^ab^
SV40	21.5 ± 1.4 ^bc^	4.0 ± 1.1 ^ac^	79.3 ± 3.0 ^cd^	104.8 ± 5.5 ^bc^	1.1 ± 0.2 ^ab^	2.6 ± 0.5 ^ab^	3.8 ± 0.7 ^ab^
B10	27.0 ± 3.7 ^c^	5.6 ± 1.3 ^c^	103.1 ± 20.0 ^d^	135.7 ± 25.0 ^c^	0.9 ± 0.0 ^a^	2.9 ± 0.3 ^ab^	3.9 ± 0.3 ^ab^
B25	27.5 ± 6.5 ^c^	5.3 ± 1.6 ^bc^	103.4 ± 14.8 ^d^	136.2 ± 22.8 ^c^	1.1 ± 0.1 ^ab^	4.0 ± 0.5 ^b^	5.2 ± 0.6 ^ab^

S10, S25, and S40 indicate 10, 25, and 40 min of exposure to steaming in the saturated oven; SV10, SV25, and SV40 indicate 10, 25, and 40 min of exposure to sous-vide in the saturated oven; B10 and B25 indicate 10 and 25 min of exposure to boiling in the hot water. Different letters within columns indicate significant differences (Tukey’s test, *p* < 0.05).

**Table 3:** Sterols and tocopherols extracted from *Cheddar* cauliflower before (fresh) and after heating under boiling (B), steaming (S), and *sous-vide* (SV). Data are expressed as dry basis (top, mg/kg dw) and wet basis (bottom, mg/kg *w*/*w*).

Sample	Campesterol	Stigmasterol	β-sitosterol	Total sterols	α-tocopherol	γ-tocopherol	Total tocopherols
	mg/kg	mg/kg	mg/kg	mg/kg	mg/kg	mg/kg	mg/kg
*Dry basis*							
Raw	140.4 ± 2.1 ^a^	19.8 ± 8.3 ^a^	585.0 ± 2.1 ^a^	745.2 ± 12.5 ^a^	8.4 ± 0.1 ^a^	17.9 ± 1.5 ^a^	28.5 ± 1.4 ^a^
S10	254.3 ± 35.9 ^abc^	42.5 ± 13.3 ^a^	1034.6 ± 106.5 ^bcd^	1331.5 ± 153.5 ^bcd^	14.5 ± 0.2 ^bc^	66.9 ± 8.5 ^b^	84.7 ± 13.8 ^b^
S25	348.8 ± 28.2 ^c^	58.1 ± 11.0 ^a^	1501.7 ± 134.0 ^e^	1908.6 ± 172.8 ^e^	15.7 ± 0.4 ^cd^	80.4 ± 5.5 ^bc^	99.7 ± 16.5 ^bc^
S40	319.8 ± 87.1 ^bc^	61.2 ± 23.9 ^a^	1370.0 ± 300.7 ^de^	1751.0 ± 411.8 ^de^	14.4 ± 0.3 ^bc^	68.9 ± 4.2 ^b^	86.8 ± 16.5 ^b^
SV10	173.7 ± 13.4 ^a^	29.4 ± 4.8 ^a^	722.8 ± 27.9 ^ab^	925.9 ± 46.1 ^ab^	10.1 ± 0.4 ^ab^	46.4 ± 3.2 ^ab^	59.8 ± 9.4 ^ab^
SV25	215.8 ± 54.1 ^ab^	63.0 ± 46.3 ^a^	990.5 ± 153.6 ^cd^	1269.3 ± 203.2 ^cd^	14.7 ± 0.4 ^bc^	65.2 ± 4.5 ^b^	83.3 ± 10.2 ^b^
SV40	306.0 ± 28.3 ^bc^	64.1 ± 31.2 ^a^	1256.6 ± 108.5 ^ce^	1626.7 ± 165.9 ^ce^	14.4 ± 0.2 ^bc^	63.1 ± 6.4 ^b^	81.0 ± 4.7 ^b^
B10	219.1 ± 10.1 ^ab^	41.1 ± 4.7 ^a^	909.4 ± 53.1 ^bc^	1169.7 ± 57.3 ^bc^	20.4 ± 0.4 ^d^	106.7 ± 11.2 ^c^	131.8 ± 19.8 ^c^
B25	718.9 ± 14.8 ^d^	147.6 ± 19.7 ^b^	2955.5 ± 92.2 ^f^	3822.0 ± 57.8 ^f^	26.6 ± 0.5 ^e^	150.7 ± 12.4 ^d^	183.4 ± 24.1 ^d^
*Wet basis*							
Raw	9.5 ± 0.5 ^a^	1.4 ± 0.6 ^a^	39.7 ± 1.8 ^ab^	50.5 ± 3.0 ^ab^	0.6 ± 0.09 ^a^	1.3 ± 0.01 ^a^	2.0 ± 0.1 ^a^
S10	13.7 ± 3.3 ^abc^	2.3 ± 0.8 ^a^	56.2 ± 13.7 ^ab^	72.1 ± 17.5 ^abc^	0.8 ± 0.07 ^a^	3.7 ± 0.01 ^b^	4.7 ± 1.8 ^b^
S25	22.1 ± 2.1 ^d^	3.7 ± 0.8 ^a^	95.3 ± 9.8 ^c^	121.2 ± 12.7 ^d^	1.0 ± 0.07 ^a^	5.1 ± 0.02 ^b^	6.3 ± 1.2 ^b^
S40	20.6 ± 5.5 ^bcd^	3.9 ± 1.5 ^a^	88.4 ± 18.8 ^c^	113.0 ± 25.8 ^cd^	0.9 ± 0.08 ^a^	4.5 ± 0.01 ^b^	5.6 ± 0.6 ^b^
SV10	11.5 ± 0.5 ^ab^	1.9 ± 0.2 ^a^	47.9 ± 0.1 ^ab^	61.3 ± 0.8 ^ab^	0.7 ± 0.08 ^a^	3.1 ± 0.02 ^ab^	4.0 ± 0.7 ^ab^
SV25	14.7 ± 3.6 ^abc^	4.3 ± 3.2 ^a^	67.5 ± 10.6 ^bc^	86.6 ± 14.2 ^bcd^	1.0 ± 0.09 ^a^	4.4 ± 0.01 ^b^	5.7 ± 0.6 ^b^
SV40	20.8 ± 1.8 ^cd^	4.3 ± 2.0 ^a^	85.3 ± 6.5 ^c^	110.5 ± 10.1 ^d^	1.0 ± 0.04 ^a^	4.3 ± 0.02 ^b^	5.5 ± 0.4 ^b^
B10	8.5 ± 0.5 ^a^	1.6 ± 0.3 ^a^	35.3 ± 1.1 ^a^	45.4 ± 1.6 ^a^	0.8 ± 0.01 ^a^	4.1 ± 0.01 ^b^	5.1 ± 0.6 ^b^
B25	23.2 ± 2.8 ^d^	4.8 ± 1.1 ^a^	95.2 ± 6.5 ^c^	123.2 ± 10.4 ^d^	0.9 ± 0.02 ^a^	5.0 ± 0.01 ^c^	6.1 ± 1.1 ^b^

S10, S25, and S40 indicate 10, 25, and 40 min of exposure to steaming in the saturated oven; SV10, SV25, and SV40 indicate 10, 25, and 40 min of exposure to sous-vide in the saturated oven; B10 and B25 indicate 10 and 25 min of exposure to boiling in the hot water. Different letters within columns indicate significant differences (Tukey’s test, *p* < 0.05).

**Table 4:** Application of stepwise regression analysis for the prediction of the concentration of sterols and tocopherols (on dry basis) that can be extracted from cauliflower after cooking by using **ERA** and **EPR** as input predictors.

	Model	Calibration Data Multiple R^2^ Pred vs. Obs	*p* (0.05)	Validation Data Multiple R^2^ Pred vs. Obs	*p* (0.05)
*Depurple*					
S	**TotSte** = 2385.37 (±462.71) − 92.24 (±29.56) ·**EPR**	0.882	0.003	0.883	0.05
S	**TotToc** = 121.96 (±29.08) − 2.04 (±0.58) · **ERA**	0.999	0.000	0.999	0.003
SV	**TotSte** = −2388.19 (±896.02) + 283.43 (±73.41) · **EPR**	0.877	0.000	0.897	0.050
SV	**TotToc** = 27.57 (±7.22) − 1.54 (±0.12) · **ERA** + 2.06 (±0.59) · **EPR**	0.988	0.000	0.978	0.010
B	**TotSte** = 118.65 (±6.44) · **EPR**	0.994	0.000	0.994	0.046
B	**TotToc** = −58.50 (±1.09) + 6.07 (±0.44) · **EPR**	0.999	0.000	0.999	0.008
*Cheddar*					
S	**TotSte** = 1257.53 (±243.53) − 82.66 (±14.52) · **ERA** + 34.78 (±10.36) ·**EPR**	0.962	0.000	0.961	0.038
S	**TotToc** = 99.07 (±9.61) − 6.35 (±0.57) · **ERA**	0.967	0.000	0.999	0.000
SV	**TotSte** = –53.51 (±17.04) · **ERA** + 72.18 (±20.81) · **EPR**	0.897	0.002	0.897	0.020
SV	**TotToc** = 49.90 (±7.15) − 5.18 (±0.37) · **ERA** + 2.79 (±0.55) · **EPR**	0.977	0.000	0.981	0.009
B	**TotSte** = 87.16 (±19.65) ·**EPR**	0.912	0.000	0.913	0.050
B	**TotToc** = 4.35 (0.34) ·**EPR**	0.975	0.000	0.969	0.043

B, S and SV indicate steaming, sous-vide and boiling, respectively **TotSte**, **TotToc** are the total amount of sterols (campesterol, β-sitosterol, and stigmasterol) and tocopherols (α-tocopherol and γ-tocopherol), respectively.

## Data Availability

The data sets supporting the results of this article are available from the corresponding author, upon reasonable request.

## Supplementary Materials

The following are available online at https://www.mdpi.com/article/10.3390/foods10091969/s1. Figure S1: LC-FLD chromatogram of tocopherols obtained after alkaline saponification or acetone extraction of boiled (10 min) Depurple cauliflower sample.Figure S2: LC-FLD chromatogram of tocopherols in raw Cheddar and Depurple cauliflowers.Figure S3: LC-FLD chromatogram of tocopherols in boiled (10 min) Cheddar and Depurple cauliflowers.Figure S4: GC/MS chromatogram of phytosterols in boiled (10 min) Cheddar cauliflower.Figure S5: GC/MS chromatogram of phytosterols from boiled (10 min) Depurple cauliflower.
