# Supplementary material for: Modeling Softening Kinetics at Cellular Scale and Phytochemicals Extractability in Cauliflower under Different Cooking Treatments
Source: Foods. 2021 Aug 24;10(9):1969. doi: 10.3390/foods10091969 (PMC8472173; doi:10.3390/foods10091969)

Figure S1: LC-FLD chromatogram of tocopherols obtained after alkaline saponification or acetone extraction of boiled (B10) *Depurple* cauliflower sample

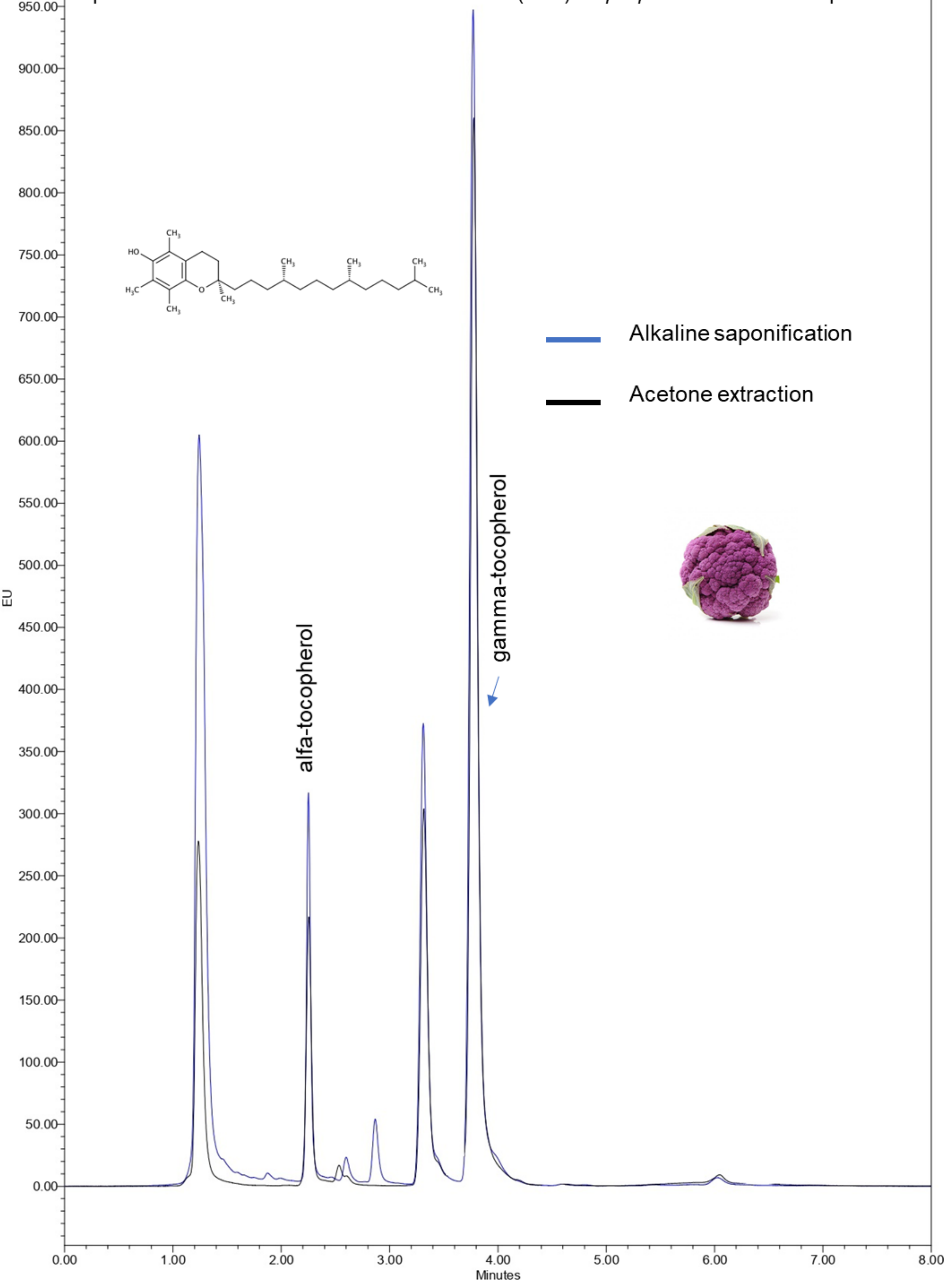

Figure S2: LC-FLD chromatogram of tocopherols in raw *Cheddar* and *Depurple* cauliflowers.

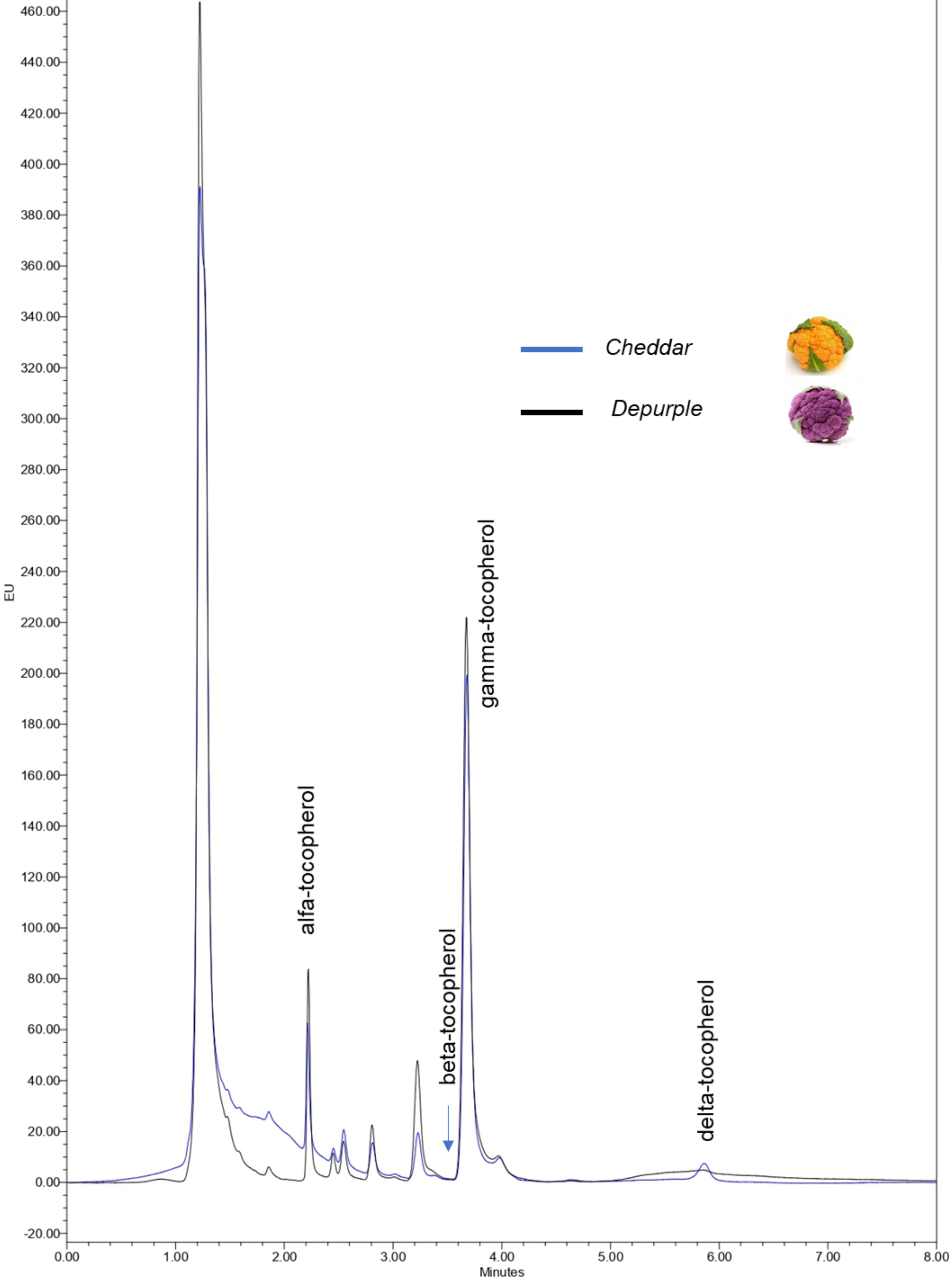

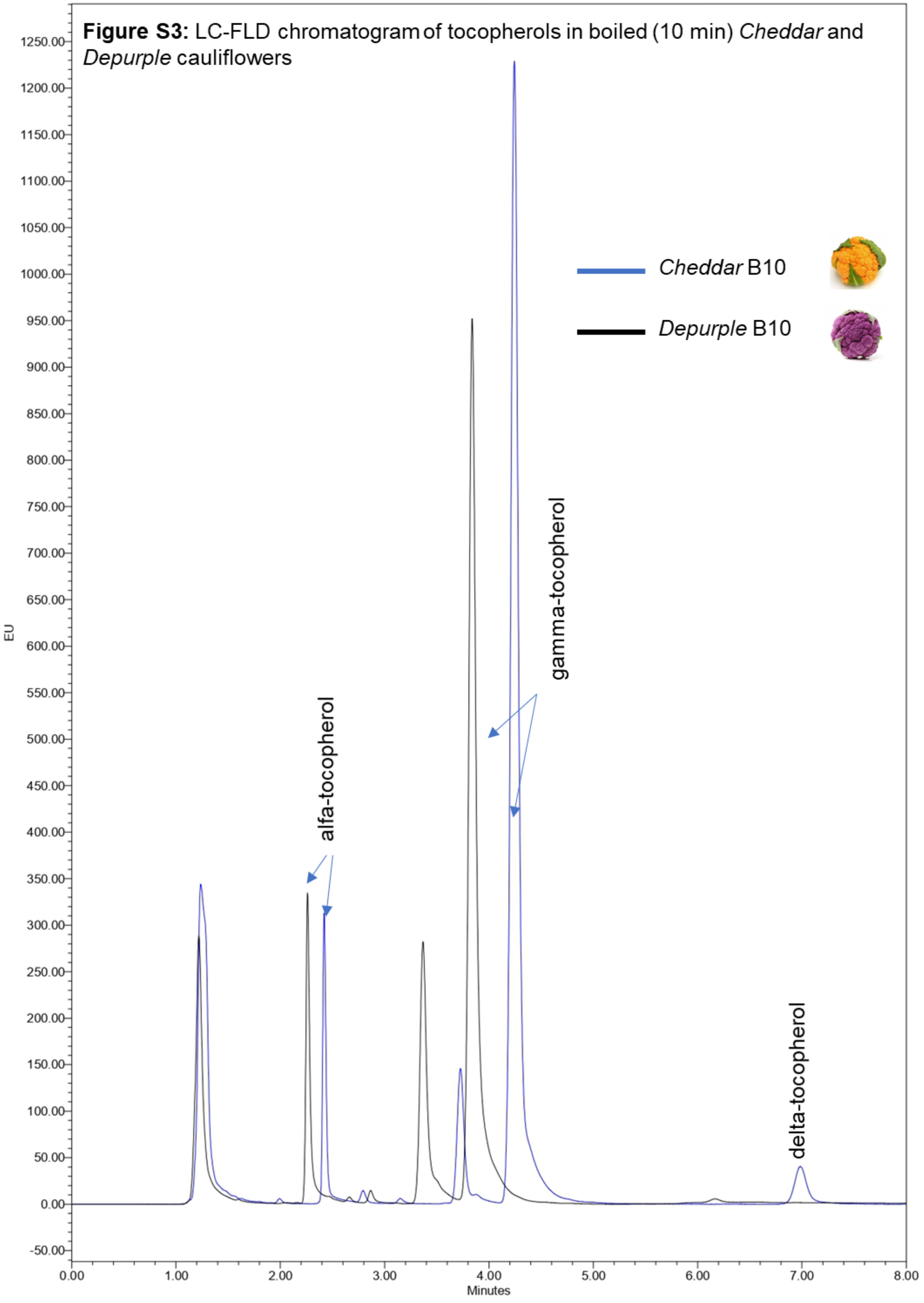

Figure S3: LC-FLD chromatogram of tocopherols in boiled (10 min) *Cheddar* and *Depurple* cauliflowers

Figure S4: GC/MS chromatogram of phytosterols in boiled (10 min) *Cheddar* cauliflower

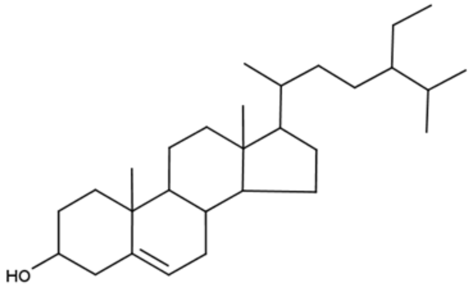

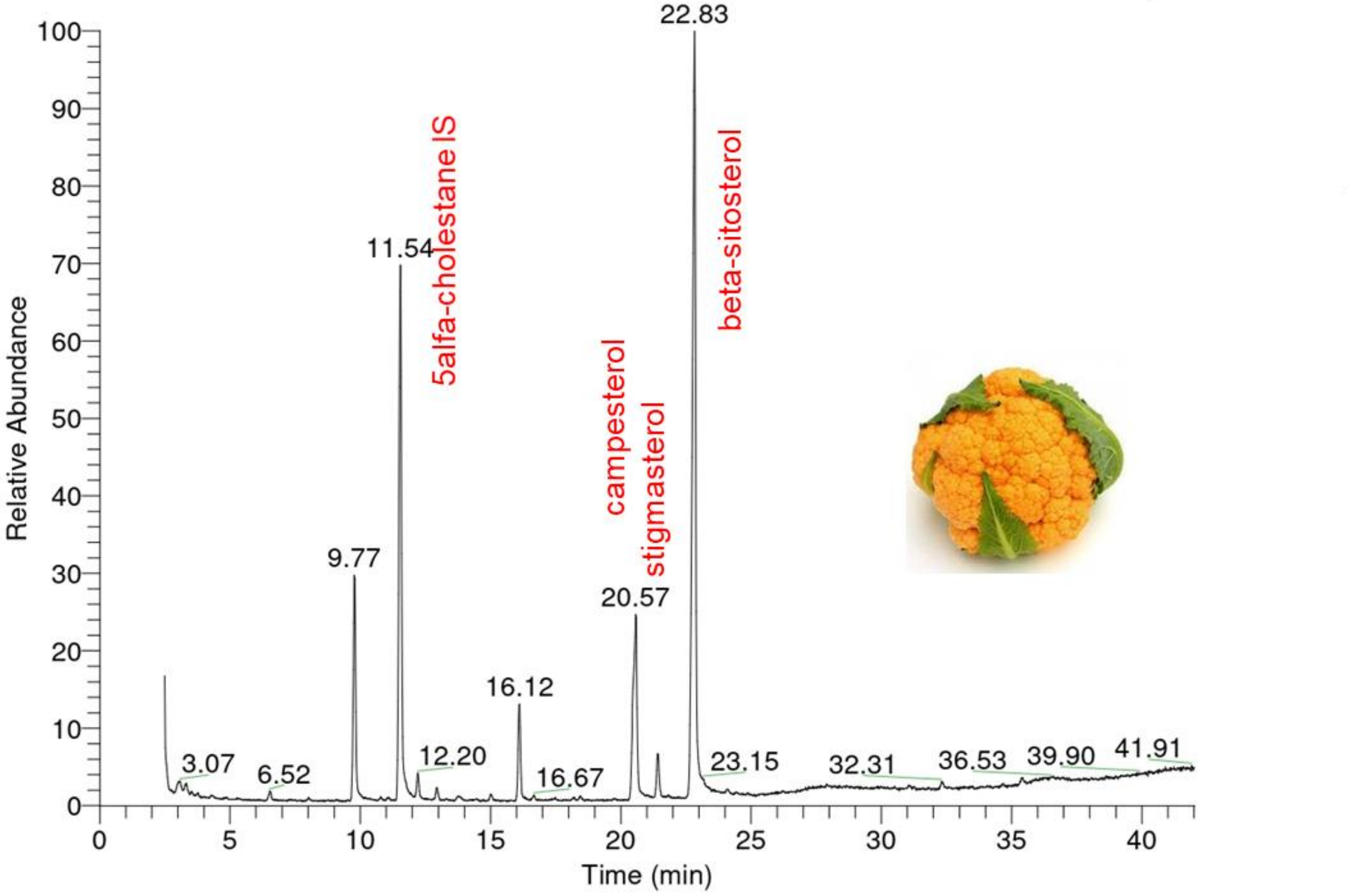

Figure S5: GC/MS chromatogram of phytosterols from boiled (10 min) *Depurple* cauliflower

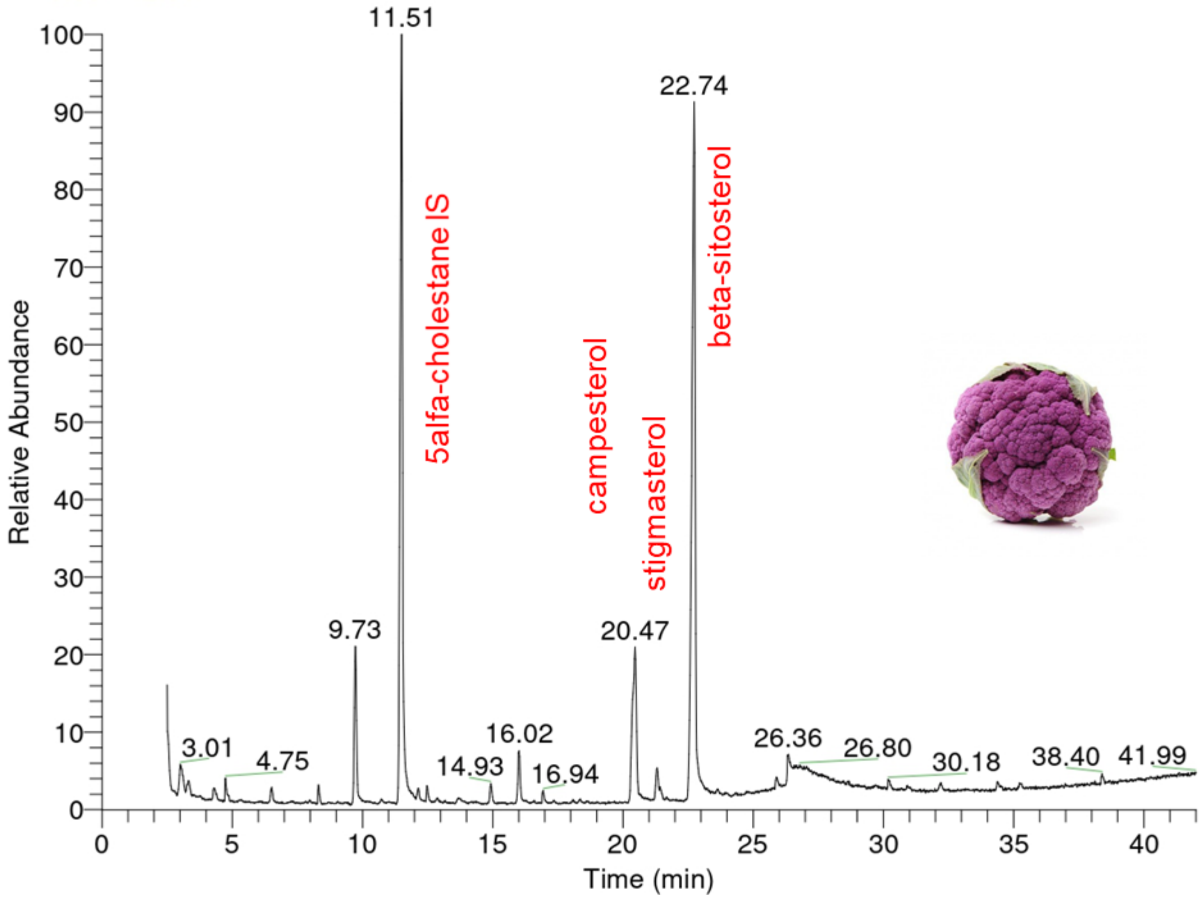

Supplement: Supplementary file 1 [file foods-10-01969-s001.zip › Supplementary data/Supplementary Data.pdf]
